# Mirage: estimation of ancestral gene-copy numbers by considering different evolutionary patterns among gene families

**DOI:** 10.1093/bioadv/vbab014

**Published:** 2021-07-30

**Authors:** Tsukasa Fukunaga, Wataru Iwasaki

**Affiliations:** 1 Waseda Institute for Advanced Study, Waseda University, Tokyo 1690051, Japan; 2 Department of Computer Science, Graduate School of Information Science and Technology, The University of Tokyo, Tokyo 1130032, Japan; 3 Department of Integrated Biosciences, Graduate School of Frontier Sciences, The University of Tokyo, Chiba 2770882, Japan; 4 Department of Biological Sciences, Graduate School of Science, The University of Tokyo, Tokyo 1130032, Japan; 5 Department of Computational Biology and Medical Sciences, Graduate School of Frontier Sciences, The University of Tokyo, Chiba 2770882, Japan; 6 Atmosphere and Ocean Research Institute, The University of Tokyo, Chiba 2770882, Japan; 7 Institute for Quantitative Biosciences, The University of Tokyo, Tokyo 1130032, Japan; 8 Collaborative Research Institute for Innovative Microbiology, The University of Tokyo, Tokyo 1130032, Japan

## Abstract

**Motivation:**

Reconstruction of gene copy number evolution is an essential approach for understanding how complex biological systems have been organized. Although various models have been proposed for gene copy number evolution, existing evolutionary models have not appropriately addressed the fact that different gene families can have very different gene gain/loss rates.

**Results:**

In this study, we developed Mirage (MIxtuRe model for Ancestral Genome Estimation), which allows different gene families to have flexible gene gain/loss rates. Mirage can use three models for formulating heterogeneous evolution among gene families: the discretized Γ model, probability distribution-free model and pattern mixture (PM) model. Simulation analysis showed that Mirage can accurately estimate heterogeneous gene gain/loss rates and reconstruct gene-content evolutionary history. Application to empirical datasets demonstrated that the PM model fits genome data from various taxonomic groups better than the other heterogeneous models. Using Mirage, we revealed that metabolic function-related gene families displayed frequent gene gains and losses in all taxa investigated.

**Availability and implementation:**

The source code of Mirage is freely available at https://github.com/fukunagatsu/Mirage.

**Supplementary information:**

Supplementary data are available at *Bioinformatics Advances* online.

## 1 Introduction

Gene gain and loss events in genomes have played essential roles in the evolutionary history of life. Complex biological systems that function through the coordination of numerous genes, e.g. metabolic pathways and signal transduction systems, have been constructed through the accumulation of such events. To answer the fundamental biological question of how such complex systems have been organized, gene-content evolutionary history has been studied with established bioinformatics methods (Fernández and Gabaldón, 2020; Hahn *et al.*, 2007; Iwasaki and Takagi, 2009; Montague *et al.*, 2014). The gene count method, which utilizes a species tree and an ortholog table, is one of the effective methods for reconstructing gene-content evolutionary history (Ames *et al.*, 2012; Cohen and Pupko, 2010; Csurös and Miklós, 2009; Hahn *et al.*, 2005; Han *et al.*, 2013; Iwasaki and Takagi, 2007; Kim and Hao, 2014; Li *et al.*, 2014, 2019; Librado *et al.*, 2012; Liu *et al.*, 2011; Rabier *et al.*, 2014; Snel *et al.*, 2002; Zamani-Dahaj *et al.*, 2016; Zwaenepoel and Van de Peer, 2020). These algorithms estimate gene content of ancestral species based on the maximum parsimony or maximum likelihood (ML) method, where the ML method is known to show better performance (Ames *et al.*, 2012; Cohen and Pupko, 2011).

In the ML method, it is important to specify which gene-content evolutionary model is adopted. The ML method first estimates evolutionary model parameters, such as gene gain and loss rates, and then the ML evolutionary history of gene content is reconstructed based on the estimated parameters. Some ML methods adopt a two-state evolutionary model and require a two-state ortholog table, which contains the presence/absence information of each ortholog group in each genome. These methods estimate whether each ortholog group existed or not at each ancestral node of the given phylogenetic tree (Cohen and Pupko, 2010; Li *et al.*, 2014). Although the two-state evolutionary model is mathematically simple, it is apparently unable to deal with gene copy number variations, which play important roles in the evolution of biological systems (Saitou and Gokcumen, 2020). The other ML methods estimate a copy number of each ortholog group at each ancestral node from an ortholog table that contains copy number information of each ortholog group in each genome.

**Fig. 1.**
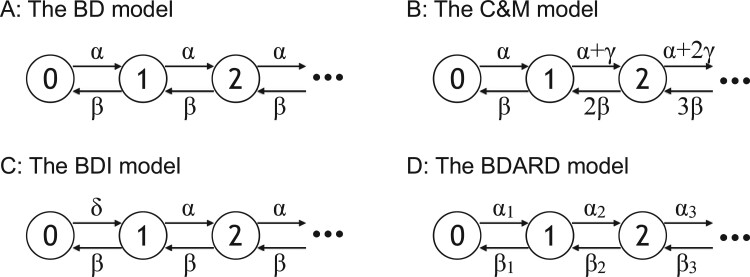
Schematic illustration of the evolutionary models. Enclosed numerals and arrows indicate gene copy numbers and the gene gain/loss events, respectively. The Greek letters denote independent rate parameters. (**A**) BD, (**B**) C&M, (**C**) BDI and (**D**) BDARD evolutionary models are shown

To date, various gene gain/loss models have been proposed for the gene copy number evolution. For example, the birth and death (BD) model is a two-parameter model, which considers only gene gain and loss parameters (Han *et al.*, 2013; Iwasaki and Takagi, 2009; Fig. 1A). Other models [the Csurös and Miklós (C&M) model and the birth, death and innovation (BDI) model] decompose the gene gain parameter into gene birth (innovation) and duplication parameters, resulting in three parameters (Ames *et al.*, 2012; Csurös and Miklós, 2009; Karev *et al.*, 2002; Fig 1B and C). A richer parameter model is the all rates different birth and death (BDARD) model, which allows all gene gain and loss parameters to be varied freely (Kim and Hao, 2014; Fig. 1D).

Another important aspect of gene-content evolution that should be considered is that different gene families have different gene gain/loss patterns (Krylov *et al.*, 2003). For example, housekeeping genes are seldom lost from genomes and thus the gene loss rates to zero copies are small, whereas antibiotic resistance genes are easily lost from genomes. Another example is olfactory receptor genes, which are prone to increase copy numbers and have exceptionally large gene gain rates. The most popular model considering the heterogeneity among gene families is the discretized Γ model, which assumes that the distribution of the evolutionary rate multipliers follows the discrete Γ distribution (Yang, 1994). Another rate multiplier heterogeneity model is the probability distribution-free (PDF) model, which directly learns evolutionary rate multipliers from an input dataset without making assumptions about the rate multiplier distribution (Kalyaanamoorthy *et al.*, 2017; Yang, 1995). These rate multiplier heterogeneity models can represent heterogeneous evolutionary rate multipliers among gene families (e.g. Kim and Hao, 2014; Librado *et al.*, 2012), but cannot represent the heterogeneity of rate patterns among gene families. For example, in the BD model with a rate multiplier heterogeneity model, the ratio between the gain and loss parameters becomes always constant among gene families. To deal with such heterogeneity, the pattern mixture (PM) model is used in molecular evolutionary analyses (Dang and Kishino, 2019; Lartillot and Philippe, 2004; Pagel and Meade, 2004; Quang *et al.*, 2008). In gene-content evolutionary analyses, the heterogeneity model has been adopted in the two-state evolutionary model (Cohen and Pupko, 2010; Li *et al.*, 2014; Spencer and Sangaralingam, 2009; Zamani-Dahaj *et al.*, 2016). Additionally, in some methods, the discretized Γ model has been used in the gene copy number evolution (Csurös and Miklós, 2009; Librado *et al.*, 2012; Mendes *et al.*, 2020). However, there have been no studies using the PDF or PM model for modeling the gene copy number evolution; in other words, existing models cannot reflect diverse gene copy number evolution patterns that depend on gene families.

In this study, we developed Mirage (MIxtuRe model for Ancestral Genome Estimation), which reconstructs a gene-content evolutionary history based on various gene gain/loss models by unsupervised classification of evolutionary patterns among gene families. We verified that Mirage can estimate both model parameters and gene-content evolutionary history with high accuracy using simulated datasets. In addition, we demonstrated that the combination of the BDARD and PM models fitted empirical datasets better than the other models. Finally, we reconstructed gene-content evolutionary histories of several taxonomic groups using Mirage and revealed that gene families involved in metabolic functions frequently experienced gene gain/loss events in all taxonomic groups investigated.

## 2 Methods

### 2.1 Input data for our method

The input data for our method are an ortholog table *D* and a phylogenetic tree *T*. *D* is a data matrix that consists of *N* species (genomes) and *L* gene families (ortholog groups). $D_{i,j}$, which is an element of the species *i* and the gene family *j* in the matrix, represents the gene copy number of *j* in *i*. The phylogenetic tree *T* is a binary rooted tree whose branches have branch lengths greater than 0. The tree has *N* leaves (external nodes), which correspond to the *N* species in the ortholog table *D*. *T* also has *N-*1 internal nodes, which correspond to the ancestral species. The reconstruction problem of gene-content evolutionary history is defined as an estimation problem of gene copy numbers (*X*) in the ancestral species for each gene family.

### 2.2 Gene-content evolutionary models

Gene-content evolution is formulated as a continuous-time Markov model, where gene copy numbers and gene gain/loss events are represented as states and state transitions, respectively. Gene gain/loss events in each gene family are assumed to have occurred independently of those in other gene families. In an infinitesimal time Δt, a gene gain/loss event of one gene is assumed to have occurred at most once. In the BD model, where the model parameters are a gene gain rate *α* and a gene loss rate *β*, transitions from a gene copy number *n* to *n* + 1 and *n-*1 occur at probabilities of αΔt and βΔt respectively, in Δt (Han *et al.*, 2013; Iwasaki and Takagi, 2009; Fig. 1A). Csurös and Miklós developed a model with three parameters: a gene acquisition rate *α*, a gene loss rate *β* and a gene duplication rate *γ* (Csurös and Miklós, 2009; Fig. 1B). By assuming that a horizontal gene transfer (HGT) is a main mechanism of gene acquisition, the C&M model defines transition rates from *n* to *n* + 1 and *n-*1 as α+nγ and nβ, respectively (note that the gene gain/loss rates change linearly with gene copy numbers). The other three-parameter model, the BDI model, utilizes a novel gene family acquisition rate *δ* in addition to a general gene gain rate *α*, and a gene loss rate *β* (Ames *et al.*, 2012; Karev *et al.*, 2002; Fig. 1C). This model is basically the same as the BD model, except that the transition rate from 0 to 1 is *δ*. The most flexible model is the BDARD model, which allows all state transition rates to be different (Kim and Hao, 2014; Fig. 1D).

For improved stability and ease of the computation, we set the maximum gene copy number as a parameter *l_max_* (i.e. gene families having copy numbers larger than *l_max_* are considered to have *l_max_* gene copies). Note that the limitation of maximum size was utilized in some previous researches (Ames *et al.*, 2012; Iwasaki and Takagi, 2007; Spencer *et al.*, 2007) and results in a finite number of parameters in the BDARD model. A large *l_max_* value introduces more parameters in the BDARD model and may cause instability of parameter estimation. Here, the number of states is $l_{\max}+1$ (0 to *l_max_*). Because *l_max_* is a user-input parameter, the user can freely set it to a reasonable value. Let R be a (*l_max_* + 1) × (*l_max_* + 1) transition rate matrix. ${\left[R\right]}_{i,j}$, which is an (*i*, *j*)-th element of R, represents the state transition rate from the state *i* to the state *j* in Δt. ${\left[R\right]}_{i,j}=0$ when |i−j|>1. We define P(y|x,R,t) as a transition probability from state *x* to *y* in time *t*. If *t* = 0, the gene copy number does not change and $P(y|x,R,0)={\left[I\right]}_{x,y}$, where *I* is the identity matrix. If $t=\Delta t,\;P(y|x,R,\Delta t)={\left[I+R\Delta t\right]}_{x,y}$, where ${\left[R\right]}_{i,i}=-\sum_{j,i\neq j}\;{\left[R\right]}_{i,j}$. Then, under the Markov process assumption, we obtain $P(y|x,R,t)={\left[\underset{n\to \infty }{\lim}{\left(I+\frac{t}{n}R\right)}^{n}\right]}_{x,y}={\left[\text{exp}(tR)\right]}_{x,y}$.

Furthermore, we allowed different gene families to have different gene gain/loss parameters. Instead of assuming that all of the *L* gene families evolve under the same transition rate matrices, these models utilize *K* transition rate matrices. Here, *K* is a user-input parameter. Each of the gene families is probabilistically assigned to *K* clusters in the framework of the mixture model. When the discretized Γ model is used, the Γ distribution is divided into *K* categories so that each category has the same probability, and calculates *r*_1_,…,*r_K_* as the mean rates of each category. Here, the Γ distribution *f*(*x*) is $\frac{\alpha^{\alpha }}{\Gamma (\alpha )}\text{exp}(-\alpha x)x^{\alpha -1}$, and the distribution is parameterized by *α*. Then, the transition parameter matrix for each cluster *k* is defined as $r_{k}R$. In addition, $\phi_{k}$, which is the probability that a gene family belongs to the category *k*, is set to $\frac{1}{K}$. When the PDF model is used, we do not assume the discrete Γ distribution for the rate multiplier distribution and directly learn *r_k_* and $\phi_{k}$ for each cluster *i* from the input data. Note that both the discretized Γ model and the PDF model use only single transition rate matrix R and thus cannot represent the heterogeneity of evolutionary patterns among gene families. Finally, when the PM model is used as the most flexible heterogeneous model, we directly introduce *K* transition rate matrices (i.e. $R_{1}$,…,$R_{K}$.) and learns $R_{k}$ and $\phi_{k}$ for each cluster *k* from the input dataset.

### 2.3 Parameter estimation and gene-content evolutionary history reconstruction algorithm

The evolutionary model parameters to be estimated for the discretized Γ model, the PDF model and the PM model are $\theta =\{\alpha ,R,\pi \},\;\{\phi ,r_{1},\ldots ,r_{K},R,\pi \}$ and $\left\{\phi ,R_{1},\ldots ,R_{K},\pi_{1},\ldots ,\pi_{K}\right\}$, respectively. Here, ϕ is a *K*-length vector that is the mixing probability of each gene-content cluster and $\pi_{k}$ is a ($l_{\max}+1$)-length vector that is the state occurrence probability of the *k*-th gene-content cluster at the root node in the phylogenetic tree. For the discretized Γ and PDF models, we assumed that all gene families follow the same distribution π. We modeled π and R as independent parameters whereas π is generally modeled as the stationary distribution of the Markov process formulated by the parameter matrix R in the DNA evolution models. This is because it is difficult to assume stationarity in the gene-content evolution (Wolf and Koonin, 2013).

The model parameters are estimated by the EM algorithm (Dempster *et al.*, 1977). The EM algorithm is an ML method for estimating parameters from observed data in statistical models that assume unobserved hidden states. In our model, the observed data are the ortholog table *D*, while the unobserved hidden states are the gene-content evolutionary history *X* and assignments of each gene family to each gene-content cluster *Z*. The EM algorithm consists of the following four steps. (1) Initialize the model parameter *θ_old_* randomly. (2) Calculate $p(X,Z|D,\theta_{\mathrm{old}})$. (3) Calculate $\theta_{\mathrm{new}}={\;\text{argmax}}_{\theta }Q(\theta ,\theta_{\mathrm{old}})$, where $Q(\theta ,\theta_{\mathrm{old}})=\sum_{X,Z}p(X,Z|D,\theta_{\mathrm{old}})\ln p(X,Z,D|\theta )$. (4) If the log-likelihood converges, terminate the EM algorithm. Otherwise, substitute *θ_new_* for *θ_old_* and return to the step (2).

Here, we describe the EM algorithm for the PM model in detail (see Supplementary Material for those of the other models). The Q function of our EM algorithm is described as follows:

$$Q(\theta ,\theta^{\mathrm{old}})=\frac{1}{L}\sum_{l,k,X\in \Omega (D_{l})}p(X,Z_{lk}|D_{l},\theta^{\mathrm{old}})\ln p(X,Z_{lk},D_{l}|\theta ),$$

where *D_l_* is the column *l* of the ortholog table *D*, $\Omega (D_{l})$ is the set of all possible gene-content evolutionary histories on *D_l_* and *Z_lk_* is an indicator variable representing whether the gene family *l* belongs to the gene-content cluster *k*. Here, for the formula of conditional probabilities,

$$\begin{array}{c} \ln p(X,Z_{lk},D_{l}|\theta )=\ln p(X|Z_{lk},\theta )+\ln p(Z_{lk}|\theta )\\ =\ln p(X|R_{k},\pi_{k})+\ln\phi_{k}. \end{array}.$$

Therefore,

$$\begin{array}{c} Q(\theta ,\theta^{\mathrm{old}})=\frac{1}{L}\sum_{l,k}p(Z_{lk}|D_{l},\theta^{\mathrm{old}})(\ln\phi_{k}+\\ \sum_{X\in \Omega (D_{l})}p(X|R_{k}^{\mathrm{old}},\pi_{k}^{\mathrm{old}})\ln p(X|R_{k},\pi_{k})). \end{array}$$

Here,

$$\begin{array}{c} p(Z_{lk}|D_{l},\theta )\propto \phi_{k}p(D_{l}|R_{k},\pi_{k}),\\ ∴p(Z_{lk}|D_{l},\theta )=\frac{\phi_{k}p(D_{l}|R_{k},\pi_{k})}{\sum_{j=1}^{K}\phi_{j}p(D_{l}|R_{j},\pi_{j})} \end{array}.$$

We describe $p(Z_{lk}|D_{l},\theta )$ as $\gamma (Z_{lk})$ for the simplicity of the notation. Based on discussion of the sufficient statistics for the phylogenetic tree model (Kiryu, 2011),

$$\begin{array}{c} p(X|R_{k},\pi_{k})=\sum_{m,i}t_{m}{\left[R_{k}\right]}_{i,i}F^{\left(m\right)}(i,X)+\\ \sum_{m,i,j}\ln(t_{m}{\left[R_{k}\right]}_{i,j})N^{\left(m\right)}(i,j,X)+\\ \sum_{i=0}^{l_{\max}}n^{\mathrm{root}}(i,X)\ln(\pi_{ki}). \end{array}$$

We assigned a distinct index to each node and *t_m_* is a branch length between the node *m* and the parent node. $F^{\left(m\right)}(i,X)$ and $N^{\left(m\right)}(i,j,X)$ are the fractional duration of the state *i* and the number of state changes from the state *i* to the state *j* on the history *X* at the branch between the node *m* and the parent node, respectively. $n^{\mathrm{root}}(i,X)$ is an indicator variable representing whether the root node takes the state *i* on the history *X*. By substituting these formulae for the Q function, we obtained the following equation:

$$\begin{array}{c} Q(\theta ,\theta^{\mathrm{old}})=\frac{1}{L}\sum_{l,k}\gamma (Z_{lk})(\ln\phi_{k}+\\ \sum_{m,i}t_{m}{\left[R_{k}\right]}_{i,i}F^{\left(m\right)}(i,D_{l},R_{k}^{\mathrm{old}},\pi_{k}^{\mathrm{old}})+\\ \sum_{m,i,j}\ln(t_{m}{\left[R_{k}\right]}_{i,j})N^{\left(m\right)}(i,j,D_{l},R_{k}^{\mathrm{old}},\pi_{k}^{\mathrm{old}})+\\ \sum_{i}n^{\mathrm{root}}(i,D_{l},R_{k}^{\mathrm{old}},\pi_{k}^{\mathrm{old}})\ln(\pi_{ki})) \end{array},$$

where $F^{\left(m\right)}(i,D_{l},R_{k}^{\mathrm{old}},\pi_{k}^{\mathrm{old}}),\;N^{\left(m\right)}(i,j,D_{l},R_{k}^{\mathrm{old}},\pi_{k}^{\mathrm{old}})$ and $n^{\mathrm{root}}(i,D_{l},R_{k}^{\mathrm{old}},\pi_{k}^{\mathrm{old}})$ are the expected values of $F^{\left(m\right)}(i,X),\;N^{\left(m\right)}(i,j,X)$, and $n^{\mathrm{root}}(i,X)$ given *D_l_*, $R_{k}^{\mathrm{old}}$ and $\pi_{k}^{\mathrm{old}}$, respectively.

In the step 2 of our EM algorithm, we calculated the values of $\gamma (Z_{lk}),\;F^{\left(m\right)}(i,D_{l},R_{k}^{\mathrm{old}},\pi_{k}^{\mathrm{old}}),\;N^{\left(m\right)}(i,j,D_{l},R_{k}^{\mathrm{old}},\pi_{k}^{\mathrm{old}})$ and $n^{\mathrm{root}}(i,D_{l},R_{k}^{\mathrm{old}},\pi_{k}^{\mathrm{old}})$ for each *k* and *l*. These expected values can be efficiently calculated using eigenvalue decompositions of the state transition probability matrices and a dynamic programming method for the phylogenetic tree *T* (Holmes and Rubin, 2002; Kiryu, 2011; Quang *et al.*, 2008). Subsequently, we found the parameter *θ* that maximized the Q function in the step 3. The details of the step 3 are described in the Supplementary Material.

We obtained the computational time complexity per iteration as follows. The most computationally expensive part of the algorithm is calculating the expected number of transitions for each branch, which is a part of dynamic programming in step 2. We need to calculate ${\left(l_{\max}+1\right)}^{4}$ expected values because the number of states is ($l_{\max}+1$). However, since we assumed ${\left[R\right]}_{i,j}=0$ when |i−j|>1, the number of transitions from the state *i* to *j* is always 0 when |i−j|>1. Therefore, the expected values that need to be counted are $O(l_{\max}^{3})$. We have to calculate the expected values for each branch, species and cluster, thus the total computational time complexity per iteration is $O(\mathrm{NLK}l_{\max}^{3})$.

After the parameter estimation, the ML evolutionary history ($\hat{X}$) is reconstructed by a dynamic programming method using the estimated parameters. The reconstruction method is similar to the Viterbi algorithm, which obtains the ML path of hidden states in the hidden Markov model, and also resembles an algorithm for the reconstruction of ancestral protein sequences (Pupko *et al.*, 2000). The details of the algorithms are described in the Supplementary Materials. We implemented the algorithms in C++, and the source code is freely available at https://github.com/fukunagatsu/Mirage.

### 2.4 Preparation of simulated datasets

We evaluated the performance of Mirage using simulated datasets. We simulated gene-content evolution for all combinations of four gain/loss models (the BD, C&M, BDI and BDARD models) and three heterogeneity models (the discretized Γ, PDF and PM models). We used a perfect binary tree with 128 leaves as the input phylogenetic tree topology and determined the branch lengths by the Yule process with a birth rate *λ* of 5.0. For the number of gene-content clusters *K* and the maximum gene family size *l_max_*, we used two sets of parameters, (*K* = 4 and *l_max_* = 3) and (*K* = 6 and *l_max_* = 5). The parameter *θ* was different for each evolutionary model, and these were described in the Supplementary Materials.

We simulated the evolution of 10 000 gene families along the input phylogenetic tree for a simulated dataset, and we constructed an ortholog table from the gene copy numbers at the leaf nodes. We prepared 10 simulated datasets, each of which consisted of an ortholog table and a phylogenetic tree.

### 2.5 Preparation of the empirical datasets

We created three empirical datasets including Archaea (domain), Micrococcales (order) and Fungi (kingdom). We used ortholog tables provided in the STRING database (Szklarczyk *et al.*, 2019) and NCBI Taxonomy for taxonomic annotation. Next, we retrieved species in the phylogenetic trees provided by the Genome Taxonomy Database release 89 (Parks *et al.*, 2018) for Archaea and Micrococcales, and those provided by the SILVA database release 111 (Yarza *et al.*, 2017; Yilmaz *et al.*, 2014) for Fungi. Then, we removed species data that were only included in either the ortholog tables or the phylogenetic trees from those datasets. In the Fungi phylogenetic tree, some species contained multiple strains. For those species, we randomly selected one strain and removed the others. Then, we reshaped the phylogenetic trees to satisfy the following three conditions: (i) a leaf of the phylogenetic trees always corresponds to a species, (ii) tree topology is binary and (iii) the distances and the phylogenetic relationships between species are the same as in the original tree. Because there were branches with branch lengths of 0 in the Fungi phylogenetic tree, we added a pseudo length 0.0001 to all tree branches. Note that the minimum branch length excluding 0 in the tree was 0.00059, which was larger than 0.0001. Finally, the Archaea, Micrococcales and Fungi datasets comprised 151 species and 11 650 gene families, 111 species and 9523 gene families, and 123 species and 34 454 gene families, respectively. The constructed datasets are freely available at https://github.com/fukunagatsu/Mirage.

### 2.6 Evaluation

The EM algorithm is guaranteed to converge to a local optimum but not to a global optimum, and thus the estimation results can depend on the initial values of the model parameters. Therefore, we estimated parameters 100 times using the EM algorithm for each dataset and each evolutionary model, and we adopted the estimation results with the largest data likelihood.

In the simulated dataset analysis, to evaluate the effect of the heterogeneity model on the performance, we investigated the performance when we changed *K*. Additionally, to assess the accuracy of presence/absence state reconstruction of the two-state model, we examined the performance when we set *l_max_* as 1. Furthermore, we evaluated the difference in the performance among various gene gain/loss models and heterogeneity models by applying these models to the datasets generated by the BDARD model with the PM model. As the evaluation criteria for the reconstructed evolutionary history, in the experiments to evaluate the effect of *K*, gene gain/loss models and heterogeneity models, we used the proportion of gene families whose gene copy numbers were correctly estimated in ancestral nodes. We also investigated the correlation coefficients between the number of gene gain/loss events for gene families in the reconstructed history and those in the true history. In the experiments to assess the performance of the two-state model, we evaluated the estimation accuracy of the presence or absence of gene families.

To evaluate the computational time, we applied Mirage to the simulated datasets under various conditions. Six factors can affect computational time: *L* (size of gene families), *N* (size of species), *K*, *l_max_*, a gain/loss model and a heterogeneity model. To estimate the influence of each factor on the computational time, we first defined the base condition and then measured the computation time by changing only one factor from the base condition. The base condition was defined as a condition that *L* = 5000, *N* = 64, *K* = 6, *l_max_* = 3, the gain/loss model is the BDARD model, and the heterogeneity model is the PM model. We measured the computational time 100 times for each condition. The computation was conducted on an Intel Xeon Gold 6130 2.1 GHz CPU with 16GB of memory.

In the empirical dataset analysis, we tested *K* values from 1 to 10 and *l_max_* values from 2 to 4. We first divided each dataset into gene families of training and test datasets, and estimated the model parameters using the training dataset only. We then calculated log-likelihoods of the test datasets based on the estimated parameters. We divided the datasets in the following three ways. For experiment 1, we randomly divided the gene families into training and test datasets at a 4:1 ratio for each dataset. Here, the species sets were common between the two datasets. For experiment 2, for each dataset, we randomly divided the species into training and test datasets at a 1:1 ratio. In this method, some gene families were shared between the training and test datasets. For experiment 3, we further processed the datasets obtained by the second method. In particular, we randomly assigned gene families shared between the training and test datasets to either of the datasets, so that no gene families were shared between the two datasets. Therefore, in this division, both the species and gene families were different between the two datasets. The numbers of gene families for each dataset in the experiments 2 and 3 are listed in Supplementary Table S1.

## 3 Results

### 3.1 Performance evaluation of Mirage based on simulated datasets

For the evaluation of the performance of Mirage, we first applied Mirage to the simulated datasets. Supplementary Figures S1 and S2 show the relative errors of the estimated model parameters. We defined the relative error as $100\times \frac{\hat{\theta }-\theta }{\theta }$, where $\hat{\theta }$ and *θ* are the estimated and true parameters, respectively. Additionally, in order to evaluate the variability of the estimation accuracy while ignoring outliers, we used interquartile range (IQR), which is defined as a difference between the 75 and 25 percentiles of relative errors. When *K* = 4 and *l_max_* = 3, the largest IQRs among the various model settings were 2.30 for R, 12.49 for π, 7.19 for ϕ, 4.18 for *r* and 3.50 for *α*. Additionally, when *K* = 6 and *l_max_* = 5, the largest IQRs among the various model settings were 5.98 for R, 94.12 for π, 15.31 for ϕ, 7.44 for *r* and 3.44 for *α*. These results show that Mirage can estimate parameters with high accuracy although the accuracy decreased when *K* and *l_max_* were large. On the other hand, in some model settings, the maximum values of the relative errors were very large. For example, in the π estimation in the PM model, the relative errors sometimes exceed 100.0, i.e. the estimated π may substantially differ from the true parameter. Such difficulty in root parameter estimation is well-known and may stem from the fact that the root node is the topologically furthest away from the observable leaf nodes (i.e. extant genomes).

When we used the same model as the one that generated the dataset for the estimation, the median of the accuracy of the reconstructed ancestral states (gene copy numbers) was more than 75% in all model settings (Supplementary Figs S3 and S4). Additionally, the median of the correlation coefficients for evaluating the estimation accuracy of numbers of gene gain/loss events was more than 97.5 in all model settings (Supplementary Fig. S3 and S4). These results show that Mirage can reconstruct the evolutionary history with high accuracy. Next, to investigate the effect of the model misspecification, we evaluated the different model from the one that generated the dataset for the estimation. We investigated whether there is a difference in accuracy between the two methods using paired *t*-tests. We used 0.05 as the original significance level, and we adjusted the value using the Bonferroni’s multiple correction, which divides the original significance level by the number of tests. When the phylogenetic mixture model was not used (i.e. *K* = 1), the reconstruction accuracy and the correlation coefficients significantly decreased in almost all cases, likely because the heterogeneity of gene-content evolution was ignored (Supplementary Fig. S3 and S4). On the other hand, when we set *K* to a larger value than the true value, we could not observe the significant increase in the reconstruction accuracy and the correlation coefficients in any cases (Supplementary Fig. S3 and S4). On the contrary, in some cases, such as the Γ model, the larger *K* value shows better performance than the true *K* value. These results mean that increasing the value of K does not significantly impact the quantitative results.

If the copy number states were ignored and only presence/absence information was considered (i.e. if incorrect estimation among the copy numbers 1, 2 and 3 was ignored), the median of the accuracy of presence/absence state reconstruction of the ancestral nodes was more than 90.0% in all model settings (Supplementary Figs S5 and S6). When the two-state model (with the phylogenetic mixture model) was applied to those cases (i.e. *l_max_* = 1), the accuracy significantly decreased depending on the model setting in many cases (Supplementary Figs S5 and S6). However, only when we used the C&M model with the Γ model for the dataset with *K* = 4 and *l_max_* = 3, the accuracy significantly increased. This result indicates that the estimation of gene copy number evolution can become effective even when only presence/absence information is reconstructed. Mirage cannot estimate parameters when users set *l_max_* to a value larger than any value contained in the dataset. As Mirage is based on the ML method, the parameters are estimated so that the probability of an event not occurring in the dataset is zero.

We also investigated the performance among various gene gain/loss models and heterogeneity models when the datasets were generated by the BDARD model with the PM model (Supplementary Fig. S7). Although we could not observe a significant difference probably because of outliers, we found that the median of the reconstruction accuracy and the correlation coefficients of the BDARD model with the PM model were larger than those of the other models.

**Fig. 2.**
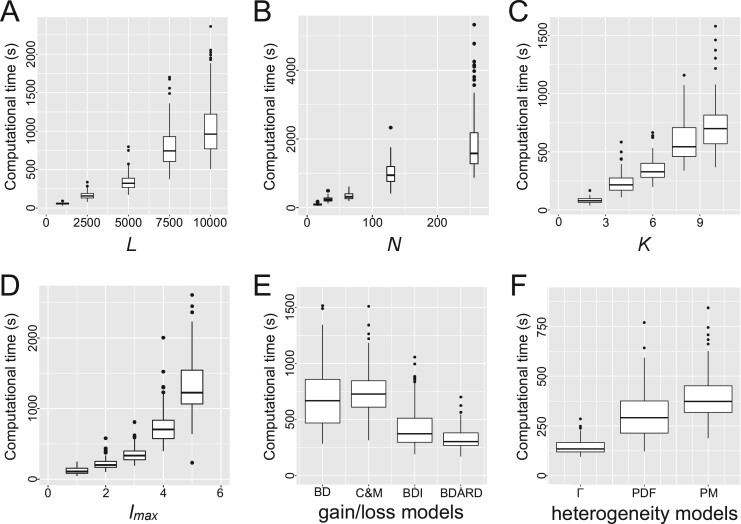
The results of the computational time evaluation on simulated datasets. The *x*-axis represents the computational time. For each figure, we have changed (**A**) *L*, (**B**) *N*, (**C**) *K*, (**D**) *l_max_*, (**E**) gain/loss models or (**F**) heterogeneity models from the base model setting.

We finally evaluated the computational time of Mirage under various conditions on the simulated datasets. We confirmed that the computational time was linearly proportional to *L*, *N* and *K* and more than linearly proportional to *l_max_* as indicated by the computational complexity analysis (Fig. 2A–D). The result about *l_max_* suggests that it is impractical to model the evolution of many copy gene families, such as olfactory receptor genes (Niimura, 2009), in the current Mirage implementation. When we changed the gain/loss models, the BDI and BDARD models were faster than the BD and C&M models (Fig. 2E). Additionally, when we changed the heterogeneity models, the PM model and the Γ model were the slowest and fastest, respectively (Fig. 2F). This result shows that the computation time increases as the complexity of heterogeneity increases.

### 3.2 Comparison of models and parameters by holdout validation based on empirical datasets

Next, we compared the effects of models and parameters by evaluating holdout performance of Mirage using empirical datasets. For the appropriate setting of *l_max_*, we first investigated the largest gene copy number among all gene families. Supplementary Fig. S8 shows cumulative relative frequency curves of the largest gene copy number in each gene family. In all datasets, the majority (80–90%) of the gene families had a maximum value of 2–4. Because large *l_max_* values require huge computation time (Fig. 2D), we tested *l_max_* values from 2 to 4 as a range of values that can be used in a case of large-scale data analysis.

**Fig. 3.**
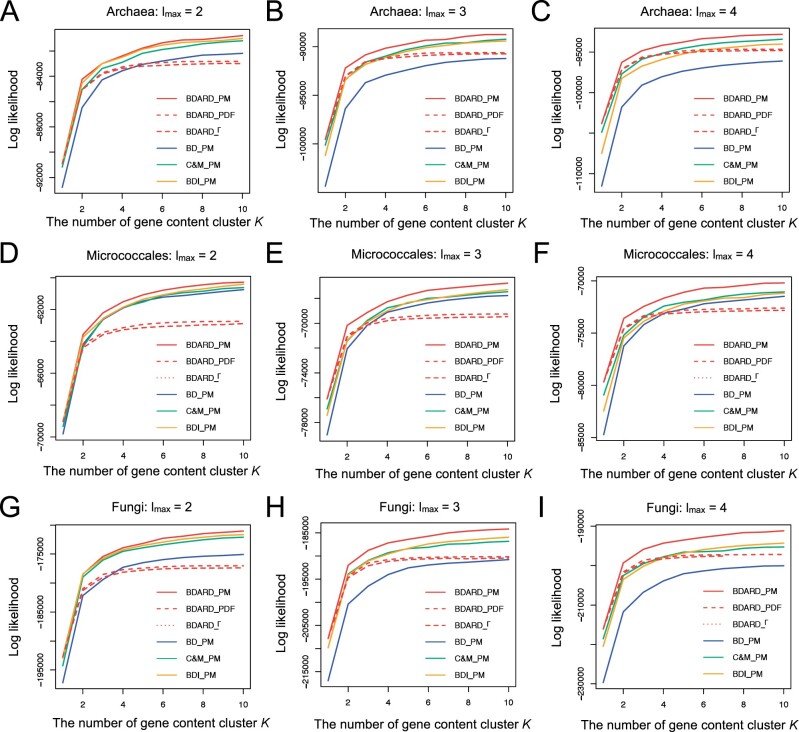
Log-likelihood values of various model settings by the holdout validation of the experiment 1. The *x*-axis and *y*-axis represent the number of gene-content clusters *K* and the log-likelihood of the test dataset, respectively. The BD, C&M, BDI and BDARD models are represented by blue, green, yellow and red lines, respectively. In addition, the PM, PDF and discretized Γ models are represented by solid, dashed and dotted lines, respectively. (**A–C**) Archaea dataset when *l_max_* was set to 2–4, (**D–F**) Micrococcales dataset when *l_max_* was set to 2–4 and (**G–I**) Fungi dataset when *l_max_* was set to 2–4

We learned the model parameters from the training datasets only under various model settings and subsequently calculated the log-likelihoods of the test datasets using the estimated parameters. Regardless of *l_max_* or the dataset used, the log-likelihood increased with the increasing number of gene-content cluster *K*, except for limited cases, likely because of convergence to local optima by the EM algorithm. In addition, the BDARD and BD models showed the best and the worst log-likelihood under the same heterogeneity model, respectively (Fig. 3). When we changed the heterogeneity model while using the BDARD model, the PM model achieved superior performances to the other models, and the PDF model showed slightly higher likelihood than the discretized Γ model. When we divided the training and test datasets in different ways (i.e. by species or by species and gene families), we obtained similar results (Supplementary Figs S9 and S10). In conclusion, the combination with the BDARD and PM models yielded gene-content evolutionary models with the largest log-likelihood values among the models we investigated.

Interestingly, although the C&M and BDI models had the same numbers of parameters, their log-likelihood values were slightly different (Fig. 3). When the Archaea or Micrococcales dataset was used and *l_max_* was 4, the C&M model exhibited a larger log-likelihood. On the other hand, when the Fungi dataset was used, the BDI model exhibited a larger log-likelihood. When $l_{\max}\geq 3$, the C&M model naturally assumes that gene duplication and loss rates change linearly with gene copies, whereas the BDI model assumes that gene duplication and loss occur at a constant rate regardless of gene copy numbers (Fig. 1). Thus, the difference likely reflects the nature of gene duplications and losses in prokaryotic and eukaryotic genomes. Specifically, the BDI model may be more suitable for eukaryotic evolutionary processes in which meiotic recombination introduces tandem gene duplications and losses, which are basically independent of gene copy numbers.

### 3.3 Analysis of estimated evolutionary model parameters

Next, we applied Mirage to each of the complete Archaea, Micrococcales and Fungi datasets. Based on the holdout validation results, we used the BDARD model with the PM model and *l_max_* = 3 for the evolutionary model. Additionally, we set *K* to 5 in order to achieve both large likelihood in the holdout validation and high interpretability thanks to the small number of *K*. Estimated model parameters are presented in Table 1, Supplementary Figure S11 and the Supplementary Data. In all datasets, the gene gain rates (${\left[R_{k}\right]}_{i,i+1}$) tended to be smaller than the gene loss rates ([${\left[R_{k}\right]}_{i,i-1}$), being consistent to a previous study (Cohen and Pupko, 2010).

**Table 1.** Estimated parameters based on the complete Archaea dataset (see Supplementary Materials for Micrococcales and Fungi datasets)

Cluster ID	ϕ	π	R
1	0.318	${\left(0.305,0.226,0.051,0.418\right)}^{T}$	$\left(\begin{array}{cccc} -0.153 & 0.153 & 0 & 0\\ 8.067 & -9.659 & 1.591 & 0\\ 0 & 11.767 & -15.246 & 3.480\\ 0 & 0 & 6.571 & -6.571 \end{array}\right)$
2	0.316	${\left(0.001,0.955,0.000,0.044\right)}^{T}$	$\left(\begin{array}{cccc} -0.017 & 0.017 & 0 & 0\\ 2.414 & -2.724 & 0.310 & 0\\ 0 & 4.540 & -5.521 & 0.981\\ 0 & 0 & 3.381 & -3.381 \end{array}\right)$
3	0.269	${\left(0.848,0.122,0.023,0.008\right)}^{T}$	$\left(\begin{array}{cccc} -0.029 & 0.029 & 0 & 0\\ 0.095 & -0.154 & 0.059 & 0\\ 0 & 0.591 & -0.881 & 0.290\\ 0 & 0 & 0.314 & -0.314 \end{array}\right)$
4	0.058	${\left(0.399,0.466,0.000,0.135\right)}^{T}$	$\left(\begin{array}{cccc} -0.207 & 0.207 & 0 & 0\\ 0.439 & -0.755 & 0.316 & 0\\ 0 & 1.792 & -2.639 & 0.847\\ 0 & 0 & 2.512 & -2.512 \end{array}\right)$
5	0.039	${\left(0.539,0.134,0.025,0.302\right)}^{T}$	$\left(\begin{array}{cccc} -0.584 & 0.584 & 0 & 0\\ 2.300 & -4.330 & 2.029 & 0\\ 0 & 5.649 & -7.394 & 1.744\\ 0 & 0 & 1.538 & -1.538 \end{array}\right)$

We next examined the evolutionary model parameters estimated for each gene-content cluster and each dataset. To quantify the frequency of gene gain/loss events occur in each cluster *k*, we calculated a normalized cluster evolutionary rate, which was $\sum_{i=0}^{l_{\max}}\pi_{i}{\left[R_{k}\right]}_{i,i}$ divided by the minimum of these values among each dataset (Supplementary Fig. S12). The maximum normalized cluster evolutionary rates were 87.4, 18.4, 18.7 for the Archaea, Micrococcales and Fungi datasets, respectively, indicating that different gene-content clusters have largely different evolutionary rates. The Archaea dataset exhibited the largest difference, where the gene-content clusters 1, 2 and 3 exhibited large, moderate and small normalized cluster evolutionary rates, respectively (Table 1). We also investigated whether specific gene functions were enriched in specific gene-content clusters. We used EGGNOG database version 4.0 for gene annotation to COG, arCOG and NOG gene families and version 3.0 for gene annotation to KOG category (Powell *et al.*, 2012, 2014). After removing ‘poorly characterized’ supercategories, we observed differences in the enriched COG supercategories among gene-content clusters (Supplementary Tables S2–S4).

### 3.4 Reconstruction of the gene-content evolutionary history

We then reconstructed the gene-content evolutionary history for each dataset using Mirage. We first counted gene gain/loss events in each gene family from the reconstructed evolutionary history (Supplementary Fig. S13). In all datasets, gene gain/loss events were rare in most gene families, whereas some gene families exceptionally frequently experienced gene/gain loss events. Supplementary Tables S5–S7 list the 20 gene families with the most frequent gene gain/loss events for each dataset. Many transposase genes were commonly found in all three datasets, whereas one gene family, COG0286 (HsdM), commonly appeared in the lists of the Archaea and Micrococcales datasets. COG0286 is annotated as a DNA methylase subunit of the type I restriction-modification system. It is reasonable that a restriction-modification system has been spread by HGT, as is well-known for the type II system (Jeltsch and Pingoud, 1996).

Finally, we investigated whether specific gene functions were enriched in the gene families with frequent gene gain/loss events. First, we examined differences in the distributions of the COG supercategories between the top 10% of gene families with frequent gene gain/loss events and entire gene families (Table 2). Based on the χ^2^ test with Bonferroni’s multiple correction, we found that the ‘metabolism’ supercategory was significantly enriched in the gene families with frequent gene gain/loss events in all datasets. Then, we analyzed which categories in the ‘metabolism’ supercategory were enriched in the different datasets (Table 3). In the Archaea dataset, gene families in categories C, ‘Energy production and conversion’, and P, ‘Inorganic ion transport and metabolism’, were the most enriched, probably reflecting the diverse ways in which Archaea obtain energy. In the Micrococcales and Fungi datasets, gene families in categories E, ‘Amino acid transport and metabolism’, G, ‘Carbohydrate transport and metabolism’, I, ‘Lipid transport and metabolism’ and Q, ‘Secondary metabolites biosynthesis, transport, and catabolism’, were highly enriched, probably reflecting rich secondary metabolism functions of those taxonomic groups.

**Table 2.** Enrichment of COG supercategories by frequent gene gain/loss events

COG supercategory	Cellular process and signaling	Information storage and processing	Metabolism
Archaea top 10%	0.231	0.199	**0.57**
Archaea whole gene families	0.243	0.27	0.486
Micrococcales top 10%	0.231	0.153	**0.617**
Micrococcales whole gene families	0.300	0.251	0.449
Fungi top 10%	0.348	0.238	**0.414**
Fungi whole gene families	0.427	0.300	0.273

The bold letter means that genes in the supercategory are likely to appear in top 10% gene families compared to the whole gene families

**Table 3.** Enrichment of COG categories in the metabolism supercategory by frequent gene gain/loss events

COG category	C	E	F	G	H	I	P	Q
Archaea top 10%	**0.146**	0.098	0.018	0.058	0.048	0.022	**0.135**	0.028
Archaea whole gene families	0.097	0.089	0.034	0.076	0.055	0.030	0.069	0.029
Micrococcales top 10%	0.071	**0.123**	0.021	**0.137**	0.040	**0.040**	**0.128**	**0.037**
Micrococcales whole gene families	0.067	0.085	0.028	0.087	0.046	0.030	0.072	0.026
Fungi top 10%	**0.056**	**0.056**	**0.025**	**0.093**	**0.026**	**0.051**	0.051	**0.026**
Fungi whole gene families	0.042	0.035	0.013	0.066	0.016	0.030	0.046	0.013

COG category symbols: C, energy production and conversion; E, amino acid transport and metabolism; F, nucleotide transport and metabolism; G, carbohydrate transport and metabolism; H, coenzyme transport and metabolism; I, lipid transport and metabolism; P, inorganic ion transport and metabolism; Q, secondary metabolites biosynthesis, transport, and catabolism. The bold letter means that genes in the category are more than 1.2 times more likely to appear in top 10% gene families compared to the whole gene families.

## 4 Discussion

In this study, we developed Mirage, which adopts heterogeneous evolutionary model among gene families for accurate ML reconstruction of gene-content evolutionary history.

We demonstrated that the combination with the BDARD and PM models achieved good performance based on empirical datasets. While the rate multiplier models, the PDF and discretized Γ models, are very frequently used in the molecular evolutionary analysis, our results show that these models may be not suitable for modeling gene copy number evolution. Molecular evolution would follow similar patterns because of physicochemical characteristics of substitutions and/or constraints due to the genetic code, whereas gene copy number evolution does not have such universal constraints and therefore may show diverse evolutionary patterns.

Whether the proposed probabilistic model is identifiable is a critical theoretical problem in statistics. Here, ‘Identifiable’ means that two different parameters always produce different probability distributions. Rhodes and Sullivant (2012) proved a theorem about a condition for identifiability in general heterogeneous evolutionary rate models. The theorem insists that heterogeneous evolutionary rate models with large *K* can be identified when the number of species is sufficiently large. However, we could not apply this theorem to our model because the assumption of the stationarity of the Markov process does not hold true. It is essential to discuss the identifiability of gene-content evolution models in the future.

Although we assumed that the input phylogenetic tree was correct, the tree topology or branch lengths may contain estimation errors. Incorrect trees would affect the estimation of ancestral gene copy numbers. The analysis of the robustness to the input tree error is important for the gene-content reconstruction analysis. Additionally, in phylogenetic tree inference, to avoid inaccurate estimation, phylogenetic relationships are often estimated not as a perfect binary tree but as a consensus tree or a phylogenetic network. Improving Mirage to accept them as input is important in mitigating the impact of estimation errors of phylogenetic trees on gene-content evolution.

The datasets in this research are unreduced datasets but not unbiased datasets because the datasets do not include OGs that are not possessed by extant organisms. Examples of these OGs are those that possessed by extinct organisms and have now been lost. The unbiased datasets have to contain these OGs because our probabilistic model can generate the all-absent patterns. Therefore, our estimation may include biases based on the unobserved patterns even if we use unreduced datasets (Cohen *et al.*, 2008; Csűrös, 2005; Felsenstein, 1992). The development of the bias correction methods for the EM algorithm is an essential future task.

The setting of the gene-content category number *K* is an important problem in Mirage. Although the Akaike Information Criterion (AIC) is a widely used estimator for model selection in phylogenetics, AIC can only be applied to statistically regular models, whose ML estimator asymptotically follows a normal distribution. Mixture models are generally nonregular models, and thus we cannot apply AIC to the heterogeneity evolution models. Another popular technique for model selection is the nonparametric Bayesian method. This method can be applied to nonregular models, but requires a lot of computation time. A practical model selection method for nonregular models is an unsolved problem in statistics, and various methods have been proposed (Fujimaki and Morinaga, 2012; Watanabe, 2013). The integration of Mirage with model selection methods for nonregular models is also a future task.

As an application of Mirage, we envision function prediction of function-unknown genes by integrating it with phylogenetic profiling to be an interesting direction. The phylogenetic profiling method predicts gene functions based on correlated occurrence patterns between genes in an ortholog table (Kensche *et al.*, 2008; Kumagai *et al.*, 2018; Sherill-Rofe *et al.*, 2019). The method generally ignores evolutionary relationships, for example by using simple mutual information as an index of correlation, and such ignorance is known to decrease prediction performance (Kensche *et al.*, 2008). Previous studies showed that the prediction performance can be improved by observing correlation patterns of gene gain/loss events in the reconstructed gene-content evolutionary history instead of gene occurrence patterns in extant species (Barker *et al.*, 2007; Moi *et al.*, 2020; Ta *et al.*, 2011). Precise reconstruction of the gene-content evolutionary history by Mirage would contribute to the improvement of the phylogenetic profiling method.

With the present Mirage implementation, it is still difficult to reconstruct gene-content evolutionary histories of all genome-sequenced species to the last universal common ancestor of life because of the huge computation time required. Thus, improving the computation time of Mirage is essential. In particular, application of the series acceleration method, which improves the convergence rate of a series, to the iteration steps of the EM algorithm seems promising. Specifically, the vector-*ϵ* acceleration technique, which does not require derivation of acceleration formula for each statistical model, may be readily applied to Mirage (Kuroda and Sakakihara, 2006). Another powerful approach would be a partitioning method, which does not use probabilistic but deterministic assignment of gene families to each gene cluster in the mixture model. This method has been widely used in molecular evolutionary analyses, but not in gene-content evolutionary analyses (Brown and Lemmon, 2007; Frandsen *et al.*, 2015; Lanfear *et al.*, 2017). Although the partitioning method can be less accurate due to the deterministic approximation, its computational efficiency would be high.

Although Mirage can model differences in the evolutionary rates among gene-content clusters, it assumes the same evolutionary rate among all branches of the phylogenetic tree. However, this assumption does not always hold true. For example, polyploidization events cause massive gene gains (Inoue *et al.*, 2015; Sriswasdi *et al.*, 2016), and parasitization events cause massive gene losses (Sun *et al.*, 2018). Moreover, heterogeneity of evolutionary rates among branches may also be caused by changes in survival strategies (Sriswasdi *et al.*, 2017) or large-scale extinction events (Wolf and Koonin, 2013). Although various programs for modeling heterogeneity among branches have been developed (Han *et al.*, 2013; Iwasaki and Takagi, 2007; Zwaenepoel and Van de Peer, 2020), there are no software that can take various gene gain/loss models and heterogeneity models into account. Therefore, the expansion of Mirage in this direction would be needed to deepen our understanding.

## Supplementary Material

vbab014_Supplementary_Data
