# Supplementary material for: Mirage: estimation of ancestral gene-copy numbers by considering different evolutionary patterns among gene families
Source: Bioinform Adv. 2021 Jul 30;1(1):vbab014. doi: 10.1093/bioadv/vbab014 (PMC9710636; doi:10.1093/bioadv/vbab014)
Supplement: vbab014_Supplementary_Data [file vbab014_supplementary_data.pdf]

Supplementary Materials for Mirage: Estimation of ancestral gene-copy numbers by considering different evolutionary patterns among gene families.

Tsukasa Fukunaga and Wataru Iwasaki

Supplementary Text

The initial parameter settings and the termination criteria of Mirage

In the step 1, we initialized the parameters $\alpha_{k,i}$ and $\beta_{k,i}$ by randomly sampling from a uniform distribution between 0.5 and 5.0. We also randomly initialized the parameters $\pi_{k,i}$ and ϕ_k to satisfy the conditions of the probability distribution. We terminated the EM algorithm in the step 4 when the increase of the log likelihood was less than 1.0 compared to the previous iteration or the number of the iteration of the step 2 and step 3 exceeded 200.

Calculation of the parameter θ that maximized the Q function for the PM model

For the BDARD model, the calculation method is as follows. We define $\alpha_{k,i}$ and $\beta_{k,i}$ as $[\boldsymbol{R_k}]_{i,i+1}$ and $[\boldsymbol{R_k}]_{i,i-1}$, respectively. Then,

$$[\boldsymbol{R_k}]_{i,i} = \begin{cases} -\alpha_{k,0} \quad (i=0) \\ -\alpha_{k,i} - \beta_{k,i} \quad (\ 1 \leq i < l_{max}\ ) \\ -\beta_{k,l_{max}} \quad (\ i = l_{max}) \end{cases}.$$

Therefore, the calculations of $\alpha_{k,i}$ and $\beta_{k,i}$ are

$$\frac{\partial Q}{\partial \alpha_{k,i}} = 0 \Leftrightarrow \alpha_{k,i} = \frac{\sum_{l=1}^{L} \gamma(Z_{lk}) \sum_m N^{(m)}(i, i+1, D_l, \boldsymbol{R_k}, \boldsymbol{\pi_k})}{\sum_{l=1}^{L} \gamma(Z_{lk}) \sum_m t_m F^{(m)}(i, D_l, \boldsymbol{R_k}, \boldsymbol{\pi_k})} \text{ and}$$

$$\frac{\partial Q}{\partial \beta_{k,i}} = 0 \Leftrightarrow \beta_{k,i} = \frac{\sum_{l=1}^{L} \gamma(Z_{lk}) \sum_m N^{(m)}(i, i-1, D_l, \boldsymbol{R_k}, \boldsymbol{\pi_k})}{\sum_{l=1}^{L} \gamma(Z_{lk}) \sum_m t_m F^{(m)}(i, D_l, \boldsymbol{R_k}, \boldsymbol{\pi_k})}.$$

In addition, $\pi_{k,i}$ and ϕ_i are calculated as follows using the method of Lagrange multiplier:

$$\frac{\partial Q}{\partial \pi_{k,i}} = 0 \Leftrightarrow \pi_{k,i} = \frac{\sum_{l=1}^{L} \gamma(Z_{lk}) n^{root}(i, D_l, \boldsymbol{R_k^{old}}, \boldsymbol{\pi_k^{old}})}{\sum_{l=1}^{L} \sum_{k=1}^{K} \gamma(Z_{lk}) n^{root}(i, D_l, \boldsymbol{R_k^{old}}, \boldsymbol{\pi_k^{old}})} \text{ and}$$

$$\frac{\partial Q}{\partial \phi_k} = 0 \Leftrightarrow \phi_k = \frac{\sum_{l=1}^{L} \gamma(Z_{lk})}{\sum_{l=1}^{L} \sum_{k=1}^{K} \gamma(Z_{lk})}.$$

For the BD model, the calculation is

$$\frac{\partial Q}{\partial \alpha_k} = 0 \Leftrightarrow \alpha_k = \frac{\sum_{l=1}^{L} \gamma(Z_{lk}) \sum_i \sum_m N^{(m)}(i, i+1, D_l, \boldsymbol{R_k}, \boldsymbol{\pi_k})}{\sum_{l=1}^{L} \gamma(Z_{lk}) \sum_i \sum_m t_m F^{(m)}(i, D_l, \boldsymbol{R_k}, \boldsymbol{\pi_k})} \text{ and}$$

$$\frac{\partial Q}{\partial \beta_k} = 0 \Leftrightarrow \beta_k = \frac{\sum_{l=1}^{L} \gamma(Z_{lk}) \sum_i \sum_m N^{(m)}(i, i-1, D_l, \boldsymbol{R_k}, \boldsymbol{\pi_k})}{\sum_{l=1}^{L} \gamma(Z_{lk}) \sum_i \sum_m t_m F^{(m)}(i, D_l, \boldsymbol{R_k}, \boldsymbol{\pi_k})}.$$

For the C&M model, the parameter β_k estimation is

$$\frac{\partial Q}{\partial \beta_k} = 0 \Leftrightarrow \beta_k = \frac{\sum_{l=1}^{L} \gamma(Z_{lk}) \sum_i \sum_m N^{(m)}(i, i-1, D_l, \boldsymbol{R_k}, \boldsymbol{\pi_k})}{\sum_{l=1}^{L} \gamma(Z_{lk}) \sum_i \sum_m t_m i F^{(m)}(i, D_l, \boldsymbol{R_k}, \boldsymbol{\pi_k})}.$$

Because we could not obtain the explicit updated formulas of α_k and γ_k for the C&M model, we updated these parameters using a gradient descent method. The learning rate was initially set to 0.1. When the newly estimated parameter was less than 0.0 or the value of the Q function based on the new parameter increased, we multiplied the learning rate by 0.1 and re-estimated the parameter. Finally, when the learning rate was less than 1.0×10^{-5}, we finished the iteration of the gradient descent method. The learning rate was initialized at each iteration of the EM algorithm.

For the BDI model, the parameter estimation is

$$\frac{\partial Q}{\partial \alpha_k} = 0 \Leftrightarrow \alpha_k = \frac{\sum_{l=1}^{L} \gamma(Z_{lk}) \sum_{i,i\neq 0} \sum_m N^{(m)}(i, i+1, D_l, \boldsymbol{R_k}, \boldsymbol{\pi_k})}{\sum_{l=1}^{L} \gamma(Z_{lk}) \sum_{i,i\neq 0} \sum_m t_m F^{(m)}(i, D_l, \boldsymbol{R_k}, \boldsymbol{\pi_k})},$$

$$\frac{\partial Q}{\partial \beta_k} = 0 \Leftrightarrow \beta_k = \frac{\sum_{l=1}^{L} \gamma(Z_{lk}) \sum_i \sum_m N^{(m)}(i, i-1, D_l, \boldsymbol{R_k}, \boldsymbol{\pi_k})}{\sum_{l=1}^{L} \gamma(Z_{lk}) \sum_i \sum_m t_m F^{(m)}(i, D_l, \boldsymbol{R_k}, \boldsymbol{\pi_k})}, \text{ and}$$

$$\frac{\partial Q}{\partial \delta_k} = 0 \Leftrightarrow \delta_k = \frac{\sum_{l=1}^{L} \gamma(Z_{lk}) \sum_m N^{(m)}(0, 1, D_l, \boldsymbol{R_k}, \boldsymbol{\pi_k})}{\sum_{l=1}^{L} \gamma(Z_{lk}) \sum_m t_m F^{(m)}(i, D_l, \boldsymbol{R_k}, \boldsymbol{\pi_k})}.$$

Parameter estimation for the PDF model and the Γ model

For the PDF model and the Γ model, the Q function is formulated as

$$Q(\theta, \theta^{old}) = \frac{1}{L} \sum_{l=1}^{L} \sum_{k=1}^{K} \gamma(Z_{lk}) \Big(\ln \phi_k + \sum_{m,i} t_m [r_k R]_{ii} F^{(m)}(i, D_l, r_k^{old}, \boldsymbol{R^{old}}, \boldsymbol{\pi^{old}}) +$$
$$\sum_{m,i,j} \ln(t_m [r_k R]_{ij}) N^{(m)}(i, j, D_l, r_k^{old}, \boldsymbol{R^{old}}, \boldsymbol{\pi^{old}}) + \sum_i n^{root}(i, D_l, r_k^{old}, \boldsymbol{R^{old}}, \boldsymbol{\pi^{old}}) \ln(\pi_i) \Big)$$

As with the calculation of the PDF model, the step 2 of the PDF model and the Γ model calculates the expected values of $F^{(m)}(i, X)$, $N^{(m)}(i, j, X)$, and $n^{root}(i, X)$ given D_l, r_k^{old}, $\boldsymbol{R^{old}}$ and $\boldsymbol{\pi^{old}}$.

Subsequently, the parameter θ that maximized the Q function should be calculated in the step 3, but we could not obtain the explicit updated formulas of r_k and $\boldsymbol{R}$ for the PDF model and the Γ model. Therefore, we estimated these two parameters alternately, i.e., we first updated the parameter $\boldsymbol{R}$ under r_k^{old}, and next updated the parameter r_k under the newly estimated parameter $\boldsymbol{R}$. This method does not maximize the Q function but is guaranteed to converge to a locally optimal solution, and is called the ECM algorithm. Finally, we normalized the parameter r_k to satisfy $\sum r_i \phi_i = 1$ by multiplying all r_i by a constant.

There are two differences between our estimation method of the PDF model and that of the Γ model. First, the parameter ϕ_k was updated in the PDF model but was fixed to $\frac{1}{K}$ in the Γ model. Second, in the Γ model, the parameter α was estimated after the termination of the ECM algorithm. Here, α was estimated so that the mean squared error between r_i estimated by the ECM algorithm and r_i derived from the Γ distribution parametrized by α was minimized.

An algorithm for reconstruction of gene content evolutionary history

We reconstructed the gene content evolutionary history based on a method roughly similar to the one presented by Pupko *et al.* This algorithm reconstructs the maximum likelihood evolutionary history. For a gene family, we define $L_x(i,k)$ as the likelihood of the maximum likelihood reconstruction of the subtree rooted at a node x when the state of the parent node of x is i and the gene family belongs to a gene content cluster k. We also define $C_x(i,k)$ as the state of the node x in that reconstruction. We do not define these values for the root node. We calculated these values from the leaf nodes to the root node by a dynamic programming method. The detail is as follows:

1. Let a be the state at a leaf node x. By the definition, $C_x(i,k)$ and $L_x(i,k)$ are always a and $P(a|i,R_k,t_x)$, respectively. Here, t_x is defined as a branch length between the node x and the parent node. We perform the calculation for all leaf nodes.

2. We consider a non-root internal node x whose children's $C_x(i,k)$ and $L_x(i,k)$ have already been calculated. Let y and z be the children of the node x. Because we would like to obtain maximum likelihood reconstruction among the possible values of j, $L_x(i,k)$ is $\max_j P(j|i,R_k,t_x) \times L_y(j,k) \times L_z(j,k)$ and $C_x(i,k)$ is j achieving the maximum. We calculate these values for all non-root internal nodes based on a dynamic programming method from leaf nodes to a root node.

3. Let y and z be the children of the root node. The likelihood of the maximum likelihood evolutionary history when the state is j and the gene content cluster is k is $\pi_k L_y(j,k) \times L_z(j,k)$. Therefore, we calculate these values for all states and gene content cluster, and select j and k maximizing the value as the reconstructed state of the root node and the gene content cluster to which the gene family belongs, respectively. Because the gene content cluster to which each gene family belongs is constant throughout the phylogenetic tree, the value of k is fixed to the value selected in this step.

4. We consider a non-root internal node x whose parent's state has already been reconstructed. When we define i as the state at the parent of the node x, we select $C_x(i,k)$ as the reconstruction of node x. We reconstruct the states for all non-root internal nodes based on a dynamic programming method from a root node to leaf nodes.

The computational time complexity is obtained as follows. For the calculation of $L_x(i,k)$, we require $O(l_{max})$ calculation. We have to calculate the values for each x, i, k, and gene family, and thus the total computational time complexity was $O(NLKl_{max}^2)$.

Supplementary Figures

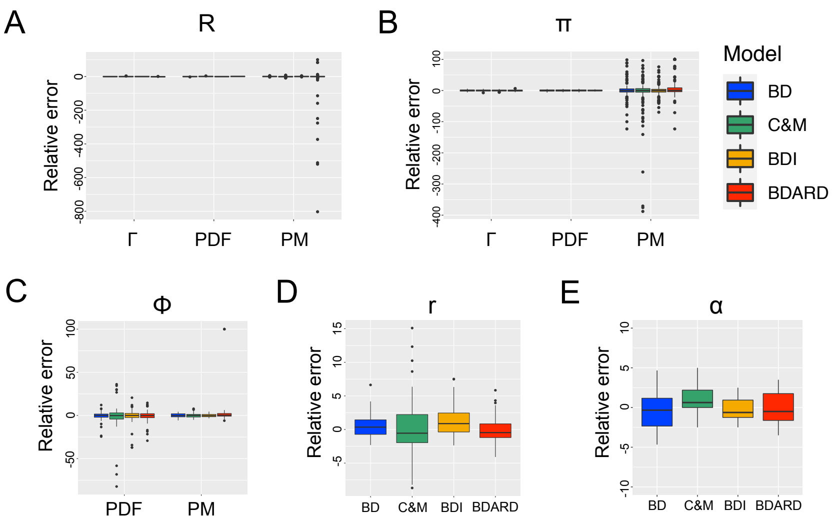

Fig. S1 Distributions of the relative errors of the estimated parameters for the simulated datasets when $K = 4$ and $l_{max} = 3$. The y-axis represents the relative error. Distributions for (A) $\boldsymbol{R}$, (B) $\boldsymbol{\pi}$, (C) $\boldsymbol{\phi}$, (D) r, and (E) α are shown. The BD, C&M, BDI and BDARD models are represented by blue, green, yellow and red bars, respectively.

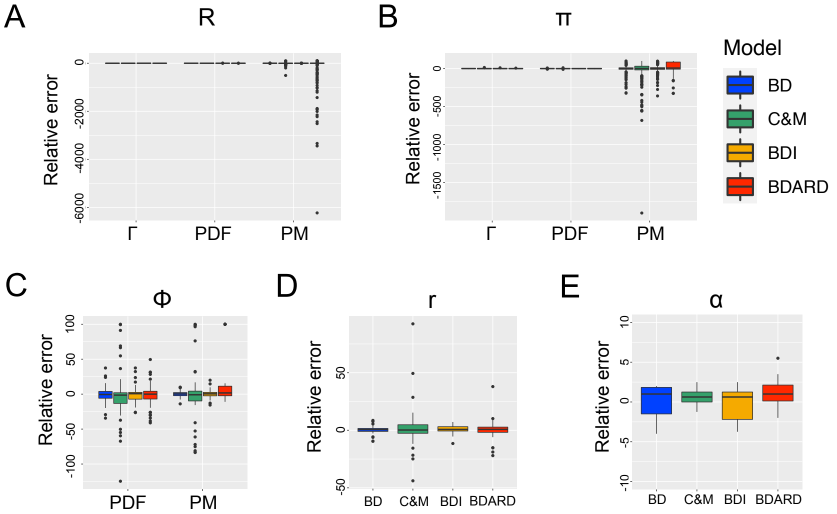

Fig. S2 Distributions of the relative errors of the estimated parameters for the simulated datasets when $K = 6$ and $l_{max} = 5$. The y-axis represents the relative error. Distributions for (A) $\boldsymbol{R}$, (B) $\boldsymbol{\pi}$, (C) $\boldsymbol{\phi}$, (D) r, and (E) α are shown. The BD, C&M, BDI and BDARD models are represented by blue, green, yellow and red bars, respectively.

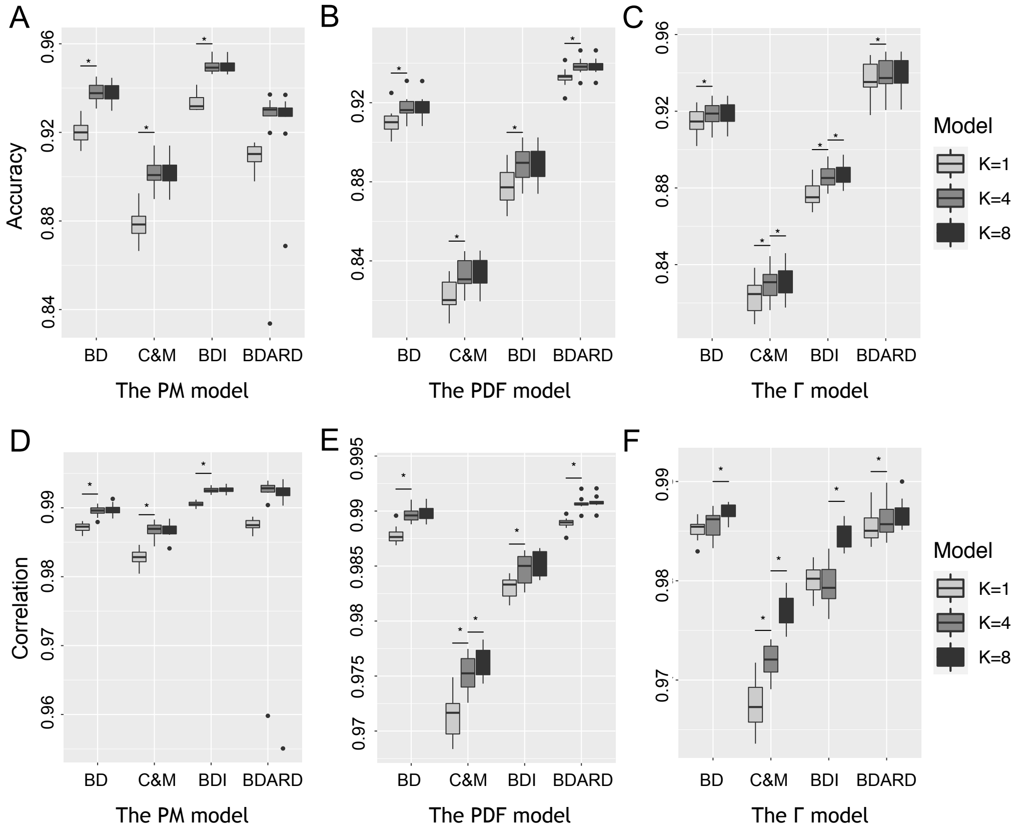

Fig. S3 Performance analysis of gene content history reconstruction for the simulated datasets when $K = 4$ and $l_{max} = 3$. (A-C) The reconstruction accuracy of the ancestral states (gene copy numbers) and (D-F) the correlation coefficients between the numbers of gene gain/loss events for gene families in the reconstructed history and those in the true history are shown. The y-axes represent the reconstruction accuracy for (A-C) and the correlation coefficients for (D-F). The model settings of $K = 1$, $K = 4$ and $K = 8$ are represented by light gray, gray and dark gray bars, respectively. * means the statistical significance under paired t-tests with Bonferroni' s multiple correction.

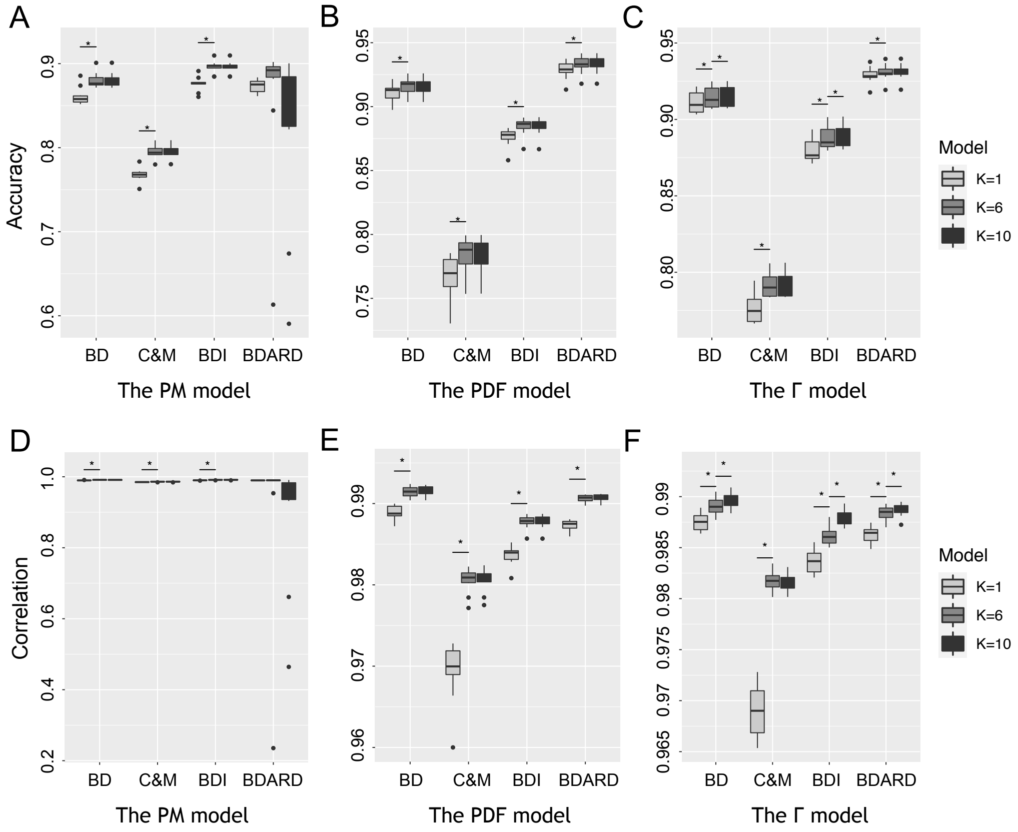

Fig. S4 Performance analysis of gene content history reconstruction for the simulated datasets when $K = 6$ and $l_{max} = 5$. (A-C) The reconstruction accuracy of the ancestral states (gene copy numbers) and (D-F) the correlation coefficients between the numbers of gene gain/loss events for gene families in the reconstructed history and those in the true history are shown. The y-axes represent the reconstruction accuracy for (A-C) and the correlation coefficients for (D-F). The model settings of $K = 1$, $K = 6$ and $K = 10$ are represented by light gray, gray and dark gray bars, respectively. * means the statistical significance under paired t-tests with Bonferroni's multiple correction.

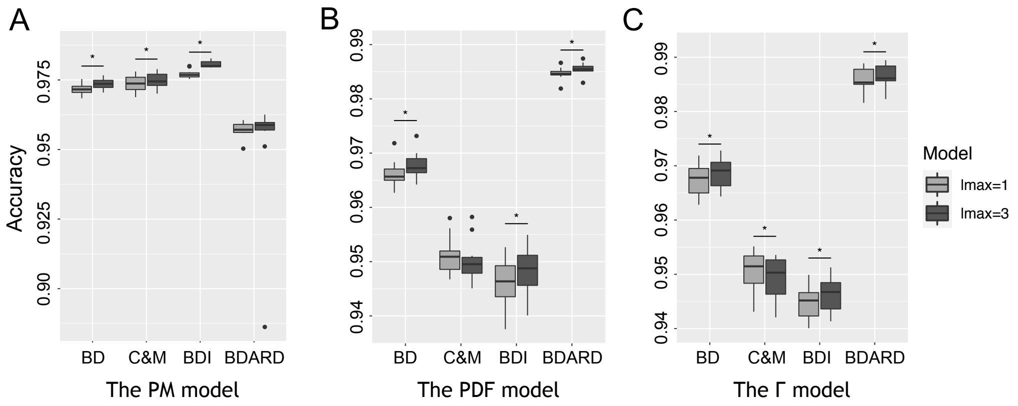

Fig. S5 Reconstruction accuracy of presence/absence state in the ancestral nodes for the simulated datasets when $K = 4$ and $l_{max} = 3$. Note that we have evaluated both cases ($l_{max} = 1$ and $l_{max} = 3$) with respect to presence/absence of gene contents. The y-axis represents the reconstruction accuracy. The model settings of $l_{max} = 1$ and $l_{max} = 3$ are represented by light gray and dark gray bars, respectively. * means the statistical significance under paired t-tests with Bonferroni' s multiple correction.

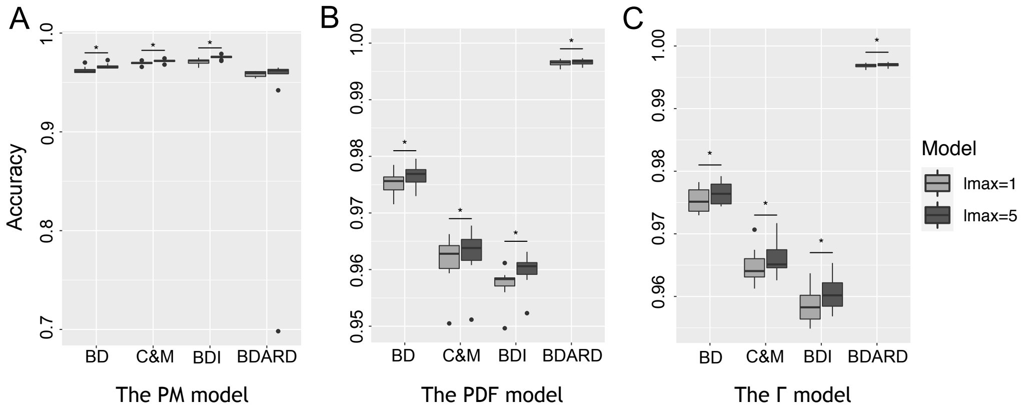

Fig. S6 Reconstruction accuracy of presence/absence state of the ancestral nodes for the simulated datasets when $K = 6$ and $l_{max} = 5$. Note that we have evaluated both cases ($l_{max} = 1$ and $l_{max} = 5$) with respect to presence/absence of gene contents.The y-axis represents the reconstruction accuracy. The model settings of $l_{max} = 1$ and $l_{max} = 5$ are represented by light gray and dark gray bars, respectively.

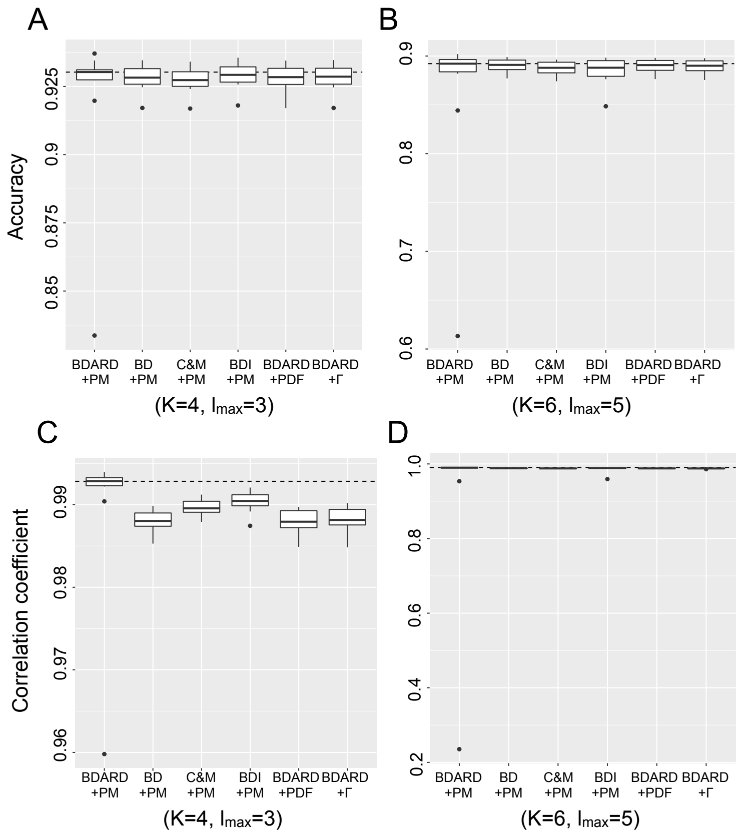

Fig. S7 Comparison analysis of gene content history reconstruction in various gene gain/loss models and heterogeneity models for the simulated datasets. We used the BDARD model with the PM model described in the Supplementary Data section as the simulation parameters. (A) The reconstruction accuracy of the ancestral states (gene copy numbers) when $K = 4$ and $l_{max} = 3$. (B) The reconstruction accuracy of the ancestral states (gene copy numbers) when $K = 6$ and $l_{max} = 4$. (C) The correlation coefficients between the numbers of gene gain/loss events for gene families in the reconstructed history and those in the true history when $K = 4$ and $l_{max} = 3$. (D) The correlation coefficients between the numbers of gene gain/loss events for gene families in the reconstructed history and those in the true history when $K = 6$ and $l_{max} = 5$. The y-axes represent the reconstruction accuracy for (A-B) and the correlation coefficients for (C-D). The dotted line represents a median value of the reconstruction accuracy (A-B) or the correlation coefficient (C-D) when using the BDARD model with the PM model.

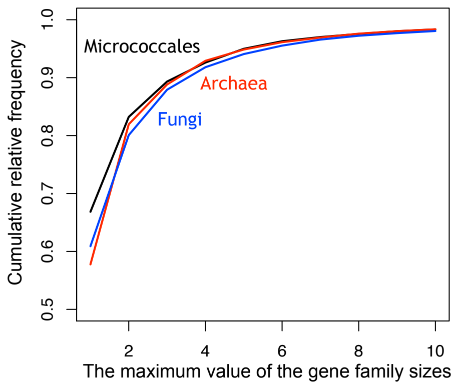

Fig. S8 Cumulative distribution curves of the maximum gene copy numbers in the empirical datasets. The x- axis and y-axis represent the maximum value of the gene copy numbers and the cumulative relative frequency, respectively. The Archaea, Micrococcales, and Fungi datasets are represented by red, black, and blue lines, respectively.

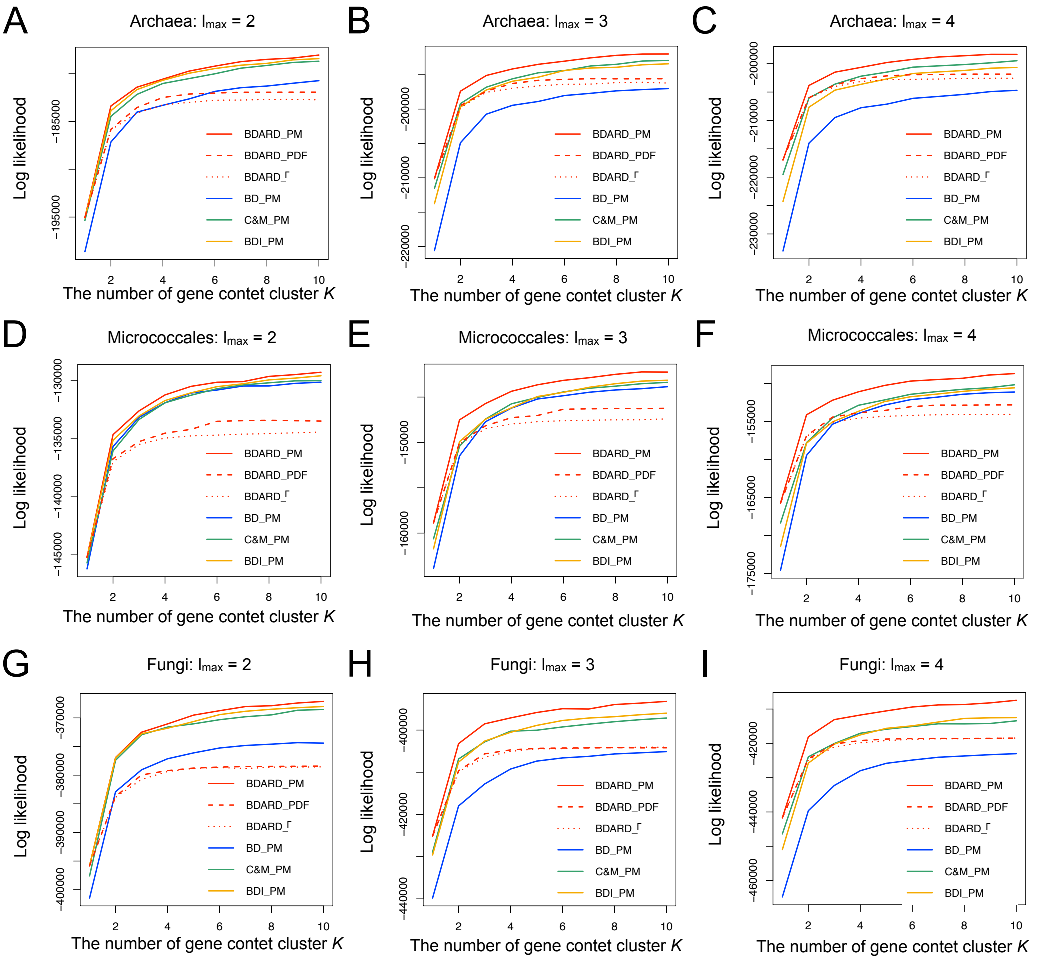

Fig. S9 Log-likelihood values of various model settings by the holdout validation of the experiment 2. The x-axis and y-axis represent the number of gene-content clusters K and the log-likelihood of the test dataset, respectively. The BD, C&M, BDI, and BDARD models are represented by blue, green, yellow, and red lines, respectively. In addition, the PM, PDF, and Γ models are represented by solid, dashed, and dotted lines, respectively. (A-C) Archaea dataset when l_{max} was set to 2-4, (D-F) Micrococcales dataset when l_{max} was set to 2-4, and (G-I) Fungi dataset when l_{max} was set to 2-4.

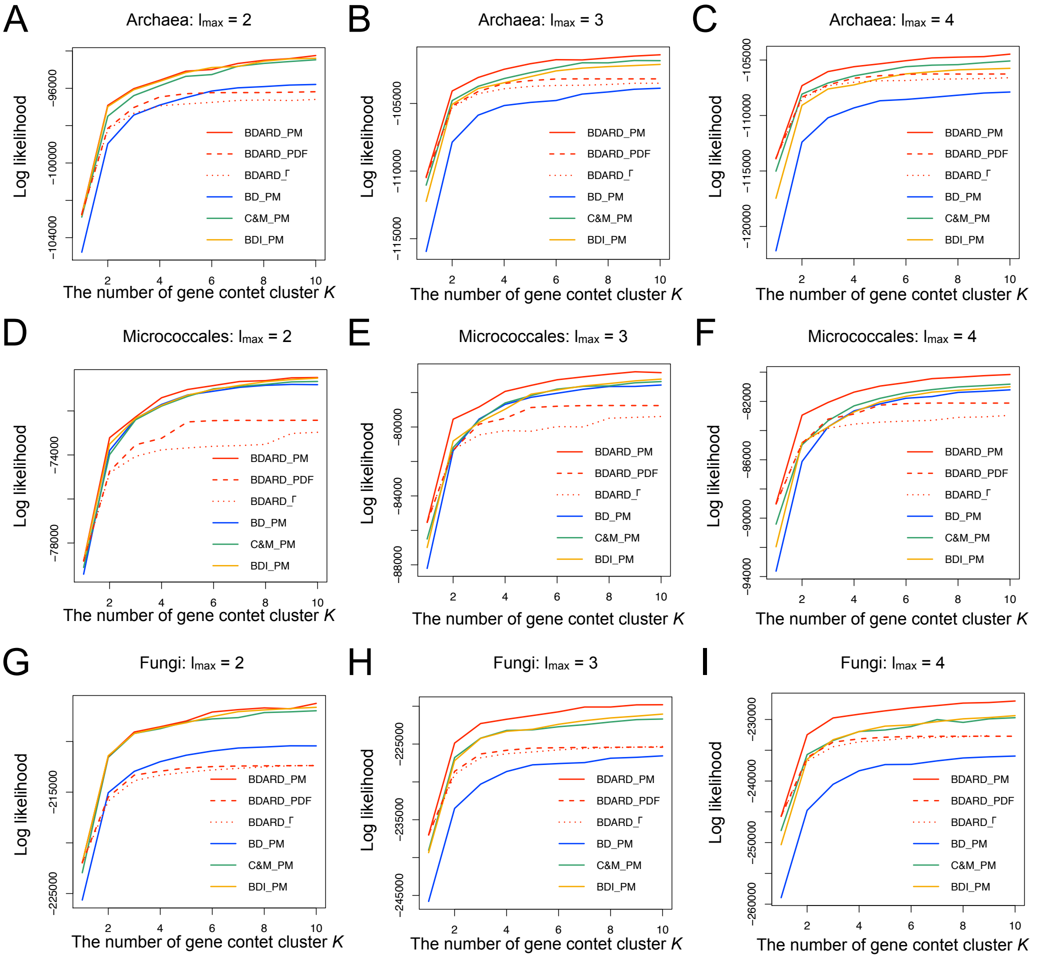

Fig. S10 Log-likelihood values of various model settings by the holdout validation of the experiment 3. The x-axis and y-axis represent the number of gene-content clusters K and the log-likelihood of the test dataset, respectively. The BD, C&M, BDI, and BDARD models are represented by blue, green, yellow, and red lines, respectively. In addition, the PM, PDF, and Γ models are represented by solid, dashed, and dotted lines, respectively. (A-C) Archaea dataset when l_{max} was set to 2-4, (D-F) Micrococcales dataset when l_{max} was set to 2-4, and (G-I) Fungi dataset when l_{max} was set to 2-4.

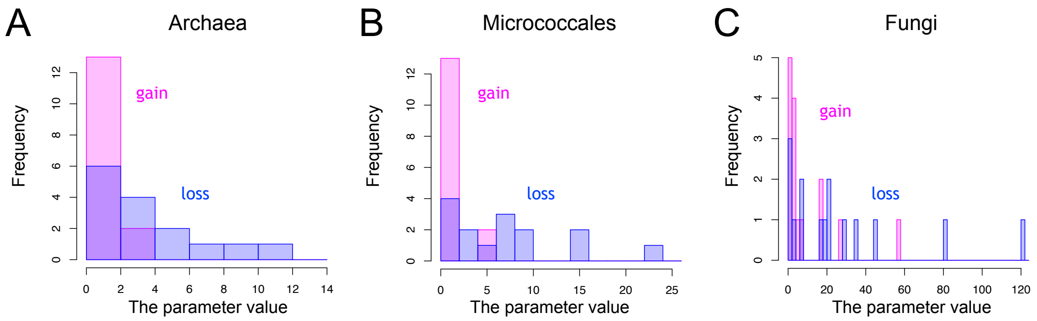

Fig. S11 The distribution of the estimated gene gain and loss rates in the empirical datasets. The x-axis and the y-axis represent the estimated parameter value and the frequency, respectively. The gene gain and loss rates are represented by red and blue bars, respectively. The panels represent the distributions for (A) the Archaea dataset, (B) the Micrococcales dataset, and (C) the Fungi dataset.

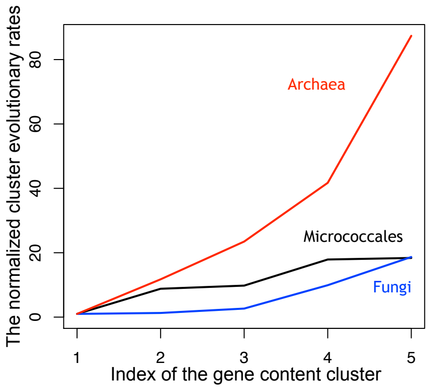

Fig. S12 The values of the normalized cluster evolutionary rates for each dataset. The x-axis and the y-axis represent the index of the gene content cluster and the normalized cluster evolutionary rates, respectively. The gene content cluster was sorted by the normalized cluster evolutionary rates. The Archaea dataset, the Micrococcales dataset, and the Fungi dataset are represented by red, black, and blue lines, respectively.

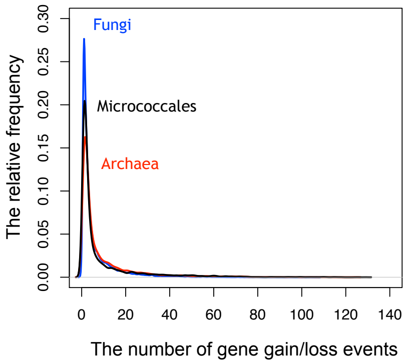

Fig. S13 The distribution of the number of gene gain/loss events for gene families in the reconstructed evolutionary history. The x-axis and the y-axis represent the number of gene gain/loss events and the relative frequency, respectively. The Archaea dataset, the Micrococcales dataset, and the Fungi dataset are represented by red, black, and blue lines, respectively.

Supplementary Tables

Table S1 The number of gene families for each dataset in the experiment 2 and 3

taxonomic group	training (experiment2)	test (experiment2)	training (experiment3)	test (experiment3)
Archaea	10425	10397	5839	5811
Micrococcales	7690	7715	4749	4774
Fungi	28462	25236	18840	15614

Table S2 The distribution of COG supercategory of the gene families for each gene content cluster in the Archaea dataset.

Cluster ID	Normalized cluster evolutionary rate	Cellular process and signaling	Information storage and processing	Metabolism
1	87.38	0.361	0.264	0.374
2	11.74	0.152	0.26	0.588
3	41.71	0.307	0.242	0.45
4	1.00	0.17	0.318	0.512
5	23.48	0.206	0.246	0.549

Table S3 The distribution of COG supercategory of the gene families for each gene content cluster in the Micrococcales dataset.

Cluster ID	Normalized cluster evolutionary rate	Cellular process and signaling	Information storage and processing	Metabolism
1	1.00	0.312	0.235	0.453
2	18.37	0.377	0.281	0.343
3	8.81	0.265	0.197	0.538
4	17.90	0.244	0.194	0.56
5	9.79	0.177	0.287	0.545

Table S4 The distribution of COG supercategory of the gene families for each gene content cluster in the Fungi dataset.

Cluster ID	Normalized cluster evolutionary rate	Cellular process and signaling	Information storage and processing	Metabolism
1	2.66	0.455	0.302	0.243
2	9.91	0.447	0.223	0.330
3	1.29	0.399	0.281	0.319
4	1.00	0.413	0.309	0.278
5	18.67	0.376	0.333	0.292

Table S5 The list of gene families with frequent gene gain/loss events for the Archaea dataset

COG ID	gene name	frequency	function
COG4743	*COG4743*	124	Uncharacterized membrane protein
COG1668	*NatB*	124	ABC-type Na+ efflux pump
COG3039	*IS5*	116	Transposase and inactivated derivatives
COG0395	*UgpE*	113	ABC-type glycerol-3-phosphate transport system
COG1487	*VapC*	113	Predicted nucleic acid-binding protein
COG3385	*InsG*	111	IS4 transposase
COG1804	*CaiB*	111	Crotonobetainyl-CoA
arCOG2330	*arCOG02330*	110	Putative signal-transducing histidine kinase / response regulator
COG1848	*COG1848*	109	Predicted nucleic acid-binding protein
COG2391	*YedE*	109	Uncharacterized membrane protein
COG1518	*Cas1*	108	CRISPR/Cas system-associated endonuclease
COG1672	*AAAA*	106	Predicted ATPase
COG3335	*Transposase*	105	Transposase
COG1216	*GT2*	105	Glycosyltransferase
COG3677	*InsA*	104	Transposase
COG0677	*WecC*	103	UDP-N-acetyl-D-mannosaminuronate dehydrogenase
COG1708	*COG1708*	102	Predicted nucleotidyltransferase
COG3316	*Rve*	102	Transposase
COG3415	*Transposase*	102	Transposase
COG0286	*HsdM*	100	Type I restriction-modification system
COG1055	*ArsB*	100	Na+/H+ antiporter NhaD

Table S6 The list of gene families with frequent gene gain/loss events for the Micrococcales dataset

COG ID	gene name	frequency	function
COG3119	*AslA*	129	Arylsulfatase A
COG3328	*IS285*	126	Transposase
COG0286	*HsdM*	125	Type I restriction-modification system
COG0402	*SsnA*	118	Cytosine/adenosine deaminase
COG3544	*COG3544*	118	Uncharacterized protein
COG0053	*FieF*	117	Divalent metal cation (Fe/Co/Zn/Cd) transporter
COG3391	*YncE*	116	DNA-binding beta-propeller fold protein
COG2334	*SrkA*	115	Ser/Thr protein kinase RdoA
COG1733	*YtfH*	115	HTH-type transcriptional regulator
COG1737	*MurR*	115	HTH-type transcriptional regulator
COG2244	*WzxE*	114	Lipid III flippase
COG3464	*COG3464*	114	Transposase
COG3176	*OlsB*	112	L-ornithine N(alpha)-acyltransferase
COG0474	*MgtA*	111	Magnesium-transporting ATPase
COG2124	*CypB*	111	Bifunctional cytochrome P450/NADPH–P450 reductase 2
COG2963	*InsE1*	110	Transposase
COG3177	*VopS*	110	Protein adenylyltransferase
COG3209	*RhsA*	110	Uncharacterized conserved protein
COG4779	*FepG*	110	Ferric enterobactin transport system permease
COG4977	*RhaS*	109	HTH-type transcriptional activator
COG0507	*RecD*	109	RecBCD enzyme subunit
COG2128	*AhpD*	109	Alkyl hydroperoxide reductase

Table S7 The list of gene families with frequent gene gain/loss events for the Fungi dataset

COG ID	gene name	frequency	function
KOG1216	*KOG1216*	107	von Willebrand factor
COG2801	*Tra5*	104	Transposase InsO
KOG3105	*KOG3105*	101	DNA-binding centromere protein B
NOG54750	*NOG54750*	101	DNA directed polymerase
NOG259057	*NOG259057*	99	Uncharacterized protein
KOG1075	*FOG*	95	Reverse transcriptase
NOG06394	*NOG06394*	93	Nitrosoguanidine resistance protein
COG1957	*Urh1*	88	Inosine-uridine nucleoside N-ribohydrolase
NOG10249	*NOG10249*	87	Uncharacterized protein
NOG258056	*NOG258056*	85	Uncharacterized protein
COG0328	*RnhA*	84	Ribonuclease HI
COG1552	*RPL40A*	84	Ribosomal protein L40E
NOG05829	*NOG05829*	84	Cloroperoxidase
COG2220	*UlaG*	83	L-ascorbate metabolism protein
COG0022	*AcoB*	81	Pyruvate/2-oxoglutarate/acetoin dehydrogenase complex
NOG258061	*NOG258061*	81	Uncharacterized protein
COG0507	*RecD*	80	ATP-dependent exoDNAse
KOG3386	*KOG3386*	80	Copper transporter
NOG252561	*NOG252561*	80	Uncharacterized protein
COG0592	*DnaN*	79	DNA polymerase III sliding clamp
COG5524	*COG5524*	79	Uncharacterized protein
KOG1773	*KOG1773*	79	Stress responsive protein
KOG4701	*KOG4701*	79	Chitinase

Supplementary Data

The simulation parameter ($K = 4$ and $l_{max} = 3$)

For the BDARD model, we defined a as a vector v_i as a vector for $([R_i]_{0,1}, [R_i]_{1,2}, [R_i]_{2,3})^T$ for simplicity of notation. In addition, we used the same values for $[R_i]_{j,j+1}$ and $[R_i]_{j-1,j}$ for any j.

(1) The BD model with the Γ model
$\alpha = 1.5$
$\pi = (0.3, 0.2, 0.2, 0.3)^T$
$\alpha = 0.7$, $\beta = 0.8$

(2) The BD model with the PDF model
$\phi = (0.5, 0.2, 0.2, 0.1)^T$
$r = (0.2, 1.0, 2.0, 3.0)^T$
$\pi = (0.3, 0.2, 0.2, 0.3)^T$
$\alpha = 0.7$, $\beta = 0.8$

(3) The BD model with the PM model
$\phi_1 = 0.5$, $\pi_1 = (0.80, 0.15, 0.04, 0.01)^T$, $\alpha_1 = 0.05$, $\beta_1 = 0.05$
$\phi_2 = 0.2$, $\pi_2 = (0.10, 0.70, 0.10, 0.10)^T$, $\alpha_2 = 0.3$, $\beta_2 = 0.5$
$\phi_3 = 0.2$, $\pi_3 = (0.45, 0.45, 0.05, 0.05)^T$, $\alpha_3 = 1.2$, $\beta_3 = 1.0$
$\phi_4 = 0.1$, $\pi_4 = (0.40, 0.10, 0.10, 0.40)^T$, $\alpha_4 = 2.5$, $\beta_4 = 2.5$

(4) The C&M model with the Γ model
$\alpha = 0.8$
$\pi = (0.3, 0.2, 0.2, 0.3)^T$
$\alpha = 0.8$, $\beta = 1.2$, $\gamma = 1.0$

(5) The C&M model with the PDF model
$\phi = (0.5, 0.2, 0.2, 0.1)^T$
$r = (0.2, 1.0, 2.0, 3.0)^T$
$\pi = (0.3, 0.2, 0.2, 0.3)^T$
$\alpha = 0.8$, $\beta = 1.2$, $\gamma = 1.0$

(6) The C&M model with the PM model
$\phi_1 = 0.5$, $\pi_1 = (0.80, 0.15, 0.04, 0.01)^T$, $\alpha_1 = 0.05$, $\beta_1 = 0.05$, $\gamma_1 = 0.05$
$\phi_2 = 0.2$, $\pi_2 = (0.10, 0.70, 0.10, 0.10)^T$, $\alpha_2 = 0.3$, $\beta_2 = 0.5$, $\gamma_2 = 0.2$
$\phi_3 = 0.2$, $\pi_3 = (0.45, 0.45, 0.05, 0.05)^T$, $\alpha_3 = 1.2$, $\beta_3 = 1.0$, $\gamma_3 = 1.0$

$\phi_4 = 0.1,\ \pi_4 = (0.40, 0.10, 0.10, 0.40)^T,\ \alpha_4 = 2.5,\ \beta_4 = 2.0,\ \gamma_4 = 1.5$

(7) The BDI model with the Γ model
$\alpha = 0.8$
$\pi = (0.3, 0.2, 0.2, 0.3)^T$
$\alpha = 0.8,\ \beta = 1.2,\ \delta = 1.0$

(8) The BDI model with the PDF model
$\phi = (0.5, 0.2, 0.2, 0.1)^T$
$r = (0.2, 1.0, 2.0, 3.0)^T$
$\pi = (0.3, 0.2, 0.2, 0.3)^T$
$\alpha = 0.8,\ \beta = 1.2,\ \delta = 1.0$

(9) The BDI model with the PM model
$\phi_1 = 0.5,\ \pi_1 = (0.80, 0.15, 0.04, 0.01)^T,\ \alpha_1 = 0.05,\ \beta_1 = 0.05,\ \delta_1 = 0.05$
$\phi_2 = 0.2,\ \pi_2 = (0.10, 0.70, 0.10, 0.10)^T,\ \alpha_2 = 0.3,\ \beta_2 = 0.5,\ \delta_2 = 0.2$
$\phi_3 = 0.2,\ \pi_3 = (0.45, 0.45, 0.05, 0.05)^T,\ \alpha_3 = 1.2,\ \beta_3 = 1.0,\ \delta_3 = 1.0$
$\phi_4 = 0.1,\ \pi_4 = (0.40, 0.10, 0.10, 0.40)^T,\ \alpha_4 = 2.5,\ \beta_4 = 2.0,\ \delta_4 = 1.5$

(10) The BDARD model with the Γ model
$\alpha = 2.0$
$\pi = (0.3, 0.3, 0.2, 0.2)^T$
$v = (0.4, 0.6, 0.8)^T$

(11) The BDARD model with the PDF model
$\phi = (0.5, 0.2, 0.2, 0.1)^T$
$r = (0.2, 1.0, 2.0, 3.0)^T$
$\pi = (0.3, 0.3, 0.2, 0.2)^T$
$v = (0.4, 0.6, 0.8)^T$

(12) The BDARD model with the PM model
$\phi_1 = 0.5,\ \pi_1 = (0.80, 0.15, 0.04, 0.01)^T,\ v_1 = (0.05, 0.05, 0.05)$
$\phi_2 = 0.2,\ \pi_2 = (0.10, 0.70, 0.10, 0.10)^T,\ v_2 = (0.20, 0.30, 0.50)$
$\phi_3 = 0.2,\ \pi_3 = (0.45, 0.45, 0.05, 0.05)^T,\ v_3 = (2.00, 0.50, 0.50)$
$\phi_4 = 0.1,\ \pi_4 = (0.40, 0.10, 0.10, 0.40)^T,\ v_4 = (2.50, 3.00, 4.50)$

The simulation parameter ($K = 6$ and $l_{max} = 5$)

For the BDARD model, we defined a as a vector v_i as a vector for $([R_i]_{0,1}, [R_i]_{1,2}, [R_i]_{2,3}, [R_i]_{2,3}, [R_i]_{3,4}, [R_i]_{4,5})^T$ for simplicity of notation. In addition, we used the same values for $[R_i]_{j,j+1}$ and $[R_i]_{j-1,j}$ for any j.

(1) The BD model with the Γ model
$\alpha = 1.5$
$\pi = (0.25, 0.15, 0.15, 0.15, 0.15, 0.15)^T$
$\alpha = 0.7$, $\beta = 0.8$

(2) The BD model with the PDF model
$\phi = (0.3, 0.2, 0.2, 0.1, 0.1, 0.1)^T$
$r = (0.1, 0.5, 1.0, 1.5, 2.0, 3.2)^T$
$\pi = (0.25, 0.15, 0.15, 0.15, 0.15, 0.15)^T$
$\alpha = 0.7$, $\beta = 0.8$

(3) The BD model with the PM model
$\phi_1 = 0.3$, $\pi_1 = (0.80, 0.10, 0.05, 0.02, 0.02, 0.01)^T$, $\alpha_1 = 0.05$, $\beta_1 = 0.05$
$\phi_2 = 0.2$, $\pi_2 = (0.10, 0.60, 0.10, 0.10, 0.050.05)^T$, $\alpha_2 = 0.3$, $\beta_2 = 0.5$
$\phi_3 = 0.2$, $\pi_3 = (0.40, 0.40, 0.05, 0.05, 0.05, 0.05)^T$, $\alpha_3 = 1.2$, $\beta_3 = 1.0$
$\phi_4 = 0.1$, $\pi_4 = (0.25, 0.15, 0.15, 0.15, 0.15, 0.15)^T$, $\alpha_4 = 2.0$, $\beta_4 = 2.0$
$\phi_5 = 0.1$, $\pi_5 = (0.40, 0.05, 0.05, 0.05, 0.05, 0.40)^T$, $\alpha_5 = 3.0$, $\beta_5 = 3.0$
$\phi_6 = 0.1$, $\pi_6 = (0.05, 0.05, 0.05, 0.05, 0.40, 0.40)^T$, $\alpha_6 = 2.0$, $\beta_6 = 3.0$

(4) The C&M model with the Γ model
$\alpha = 0.8$
$\pi = (0.25, 0.15, 0.15, 0.15, 0.15, 0.15)^T$
$\alpha = 0.8$, $\beta = 1.2$, $\gamma = 1.0$

(5) The C&M model with the PDF model
$\phi = (0.3, 0.2, 0.2, 0.1, 0.1, 0.1)^T$
$r = (0.1, 0.5, 1.0, 1.5, 2.0, 3.2)^T$
$\pi = (0.25, 0.15, 0.15, 0.15, 0.15, 0.15)^T$
$\alpha = 0.8$, $\beta = 1.2$, $\gamma = 1.0$

(6) The C&M model with the PM model
$\phi_1 = 0.3$, $\pi_1 = (0.80, 0.10, 0.05, 0.02, 0.02, 0.01)^T$, $\alpha_1 = 0.05$, $\beta_1 = 0.05$, $\gamma_1 = 0.05$
$\phi_2 = 0.2$, $\pi_2 = (0.10, 0.60, 0.10, 0.10, 0.05, 0.05)^T$, $\alpha_2 = 0.3$, $\beta_2 = 0.5$, $\gamma_2 = 0.2$
$\phi_3 = 0.2$, $\pi_3 = (0.40, 0.40, 0.05, 0.05, 0.05, 0.05)^T$, $\alpha_3 = 1.2$, $\beta_3 = 1.0$, $\gamma_3 = 1.0$

$\phi_4 = 0.1,\ \pi_4 = (0.25, 0.15, 0.15, 0.15, 0.15, 0.15)^T,\ \alpha_4 = 2.0,\ \beta_4 = 1.5,\ \gamma_4 = 1.5$
$\phi_5 = 0.1,\ \pi_5 = (0.40, 0.05, 0.05, 0.05, 0.05, 0.40)^T,\ \alpha_5 = 3.0,\ \beta_5 = 2.0,\ \gamma_5 = 2.0$
$\phi_6 = 0.1,\ \pi_6 = (0.05, 0.05, 0.05, 0.05, 0.40, 0.40)^T,\ \alpha_6 = 2.0,\ \beta_6 = 2.5,\ \gamma_6 = 2.5$

(7) The BDI model with the Γ model
$\alpha = 0.8$
$\pi = (0.25, 0.15, 0.15, 0.15, 0.15, 0.15)^T$
$\alpha = 0.8,\ \beta = 1.2,\ \delta = 1.0$

(8) The BDI model with the PDF model
$\phi = (0.3, 0.2, 0.2, 0.1, 0.1, 0.1)^T$
$r = (0.1, 0.5, 1.0, 1.5, 2.0, 3.2)^T$
$\pi = (0.25, 0.15, 0.15, 0.15, 0.15, 0.15)^T$
$\alpha = 0.8,\ \beta = 1.2,\ \delta = 1.0$

(9) The BDI model with the PM model
$\phi_1 = 0.3,\ \pi_1 = (0.80, 0.10, 0.05, 0.02, 0.02, 0.01)^T,\ \alpha_1 = 0.05,\ \beta_1 = 0.05,\ \delta_1 = 0.05$
$\phi_2 = 0.2,\ \pi_2 = (0.10, 0.60, 0.10, 0.10, 0.05, 0.05)^T,\ \alpha_2 = 0.3,\ \beta_2 = 0.5,\ \delta_2 = 0.2$
$\phi_3 = 0.2,\ \pi_3 = (0.40, 0.40, 0.05, 0.05, 0.05, 0.05)^T,\ \alpha_3 = 1.2,\ \beta_3 = 1.0,\ \delta_3 = 1.0$
$\phi_4 = 0.1,\ \pi_4 = (0.25, 0.15, 0.15, 0.15, 0.15, 0.15)^T,\ \alpha_4 = 2.0,\ \beta_4 = 1.5,\ \delta_4 = 1.5$
$\phi_5 = 0.1,\ \pi_5 = (0.40, 0.05, 0.05, 0.05, 0.05, 0.40)^T,\ \alpha_5 = 3.0,\ \beta_5 = 2.0,\ \delta_5 = 2.0$
$\phi_6 = 0.1,\ \pi_6 = (0.05, 0.05, 0.05, 0.05, 0.40, 0.40)^T,\ \alpha_6 = 2.0,\ \beta_6 = 2.5,\ \delta_6 = 2.5$

(10) The BDARD model with the Γ model
$\alpha = 2.0$
$\pi = (0.25, 0.15, 0.15, 0.15, 0.15, 0.15)^T$
$v = (0.2, 0.4, 0.6, 0.9, 1.2)^T$

(11) The BDARD model with the PDF model
$\phi = (0.3, 0.2, 0.2, 0.1, 0.1, 0.1)^T$
$r = (0.1, 0.5, 1.0, 1.5, 2.0, 3.2)^T$
$\pi = (0.25, 0.15, 0.15, 0.15, 0.15, 0.15)^T$
$v = (0.2, 0.4, 0.6, 0.9, 1.2)^T$

(12) The BDARD model with the PM model
$\phi_1 = 0.3,\ \pi_1 = (0.80, 0.10, 0.05, 0.02, 0.02, 0.01)^T,\ v_1 = (0.05, 0.05, 0.05, 0.05, 0.05)$
$\phi_2 = 0.2,\ \pi_2 = (0.10, 0.60, 0.10, 0.10, 0.05, 0.05)^T,\ v_2 = (0.10, 0.20, 0.30, 0.40, 0.50)$
$\phi_3 = 0.2,\ \pi_3 = (0.40, 0.40, 0.05, 0.05, 0.05, 0.05)^T,\ v_3 = (2.00, 0.50, 0.50, 0.50, 0.50)$
$\phi_4 = 0.1,\ \pi_4 = (0.25, 0.15, 0.15, 0.15, 0.15, 0.15)^T,\ v_4 = (2.00, 2.00, 2.00, 2.00, 2.00)$
$\phi_5 = 0.1,\ \pi_5 = (0.40, 0.05, 0.05, 0.05, 0.05, 0.40)^T,\ v_5 = (2.00, 2.50, 3.00, 3.50, 4.00)$

$\phi_6 = 0.1$, $\pi_6 = (0.05, 0.05, 0.05, 0.05, 0.40, 0.40)^T$, $v_6 = (0.10, 0.10, 0.10, 0.10, 3.00)$

The estimated parameter for the Micrococcales dataset

$$\phi_1 = 0.609,\ \pi_1 = (0.583, 0.232, 0.000, 0.124)^T,\ R_1 = \begin{pmatrix} -0.020 & 0.020 & 0 & 0 \\ 9.998 & -10.953 & 0.955 & 0 \\ 0 & 23.368 & -25.037 & 1.670 \\ 0 & 0 & 15.169 & -15.169 \end{pmatrix}$$

$$\phi_2 = 0.181,\ \pi_2 = (0.827, 0.105, 0.029, 0.038)^T,\ R_2 = \begin{pmatrix} -0.095 & 0.095 & 0 & 0 \\ 1.360 & -1.421 & 0.061 & 0 \\ 0 & 0.685 & -1.230 & 0.615 \\ 0 & 0 & 0.453 & -0.453 \end{pmatrix}$$

$$\phi_3 = 0.086,\ \pi_3 = (0.367, 0.292, 0.135, 0.207)^T,\ R_3 = \begin{pmatrix} -1.059 & 1.059 & 0 & 0 \\ 2.149 & -2.890 & 0.741 & 0 \\ 0 & 4.202 & -5.580 & 1.379 \\ 0 & 0 & 2.482 & -2.482 \end{pmatrix}$$

$$\phi_4 = 0.067,\ \pi_4 = (0.054, 0.660, 0.000, 0.286)^T,\ R_4 = \begin{pmatrix} -0.614 & 0.614 & 0 & 0 \\ 0.130 & -0.359 & 0.229 & 0 \\ 0 & 6.120 & -7.125 & 1.004 \\ 0 & 0 & 8.754 & -8.754 \end{pmatrix}$$

$$\phi_5 = 0.058,\ \pi_5 = (0.462, 0.000, 0.000, 0.537)^T,\ R_5 = \begin{pmatrix} -1.944 & 1.944 & 0 & 0 \\ 6.406 & -11.768 & 5.362 & 0 \\ 0 & 15.093 & -20.138 & 5.045 \\ 0 & 0 & 7.757 & -7.757 \end{pmatrix}$$

The estimated parameter for the Fungi dataset

$$\phi_1 = 0.534,\ \pi_1 = (0.966, 0.033, 0.001, 0.000)^T,\ R_1 = \begin{pmatrix} -0.808 & 0.808 & 0 & 0 \\ 44.159 & -60.783 & 16.624 & 0 \\ 0 & 120.758 & -178.152 & 57.394 \\ 0 & 0 & 81.525 & -81.525 \end{pmatrix}$$

$$\phi_2 = 0.271,\ \pi_2 = (0.893, 0.073, 0.008, 0.026)^T,\ R_2 = \begin{pmatrix} -0.354 & 0.354 & 0 & 0 \\ 6.618 & -7.853 & 1.235 & 0 \\ 0 & 21.577 & -25.557 & 3.980 \\ 0 & 0 & 0.782 & -0.782 \end{pmatrix}$$

$$\phi_3 = 0.111,\ \pi_3 = (0.708, 0.178, 0.079, 0.035)^T,\ R_3 = \begin{pmatrix} -0.292 & 0.292 & 0 & 0 \\ 1.665 & -2.830 & 1.164 & 0 \\ 0 & 2.892 & -6.327 & 3.435 \\ 0 & 0 & 6.230 & -6.230 \end{pmatrix}$$

$$\phi_4 = 0.045,\ \pi_4 = (0.831, 0.002, 0.152, 0.015)^T,\ R_4 = \begin{pmatrix} -3.148 & 3.148 & 0 & 0 \\ 16.272 & -23.744 & 7.472 & 0 \\ 0 & 35.932 & -52.694 & 16.762 \\ 0 & 0 & 19.097 & -19.097 \end{pmatrix}$$

$$\phi_5 = 0.039,\ \pi_5 = (0.411, 0.323, 0.193, 0.072)^T,\ R_5 = \begin{pmatrix} -27.535 & 27.535 & 0 & 0 \\ 1.580 & -3.669 & 2.089 & 0 \\ 0 & 28.536 & -33.887 & 5.351 \\ 0 & 0 & 21.795 & -21.795 \end{pmatrix}$$
